# The Impact of Different Types of Physical Effort on the Expression of Selected Chemokine and Interleukin Receptor Genes in Peripheral Blood Cells

**DOI:** 10.3390/cells12081119

**Published:** 2023-04-09

**Authors:** Robert Nowak, Alicja Trzeciak-Ryczek, Andrzej Ciechanowicz, Andrzej Brodkiewicz, Elżbieta Urasińska, Dorota Kostrzewa-Nowak

**Affiliations:** 1 Institute of Physical Culture Sciences, University of Szczecin, 17C Narutowicza St., 70-240 Szczecin, Poland; robert.nowak@usz.edu.pl; 2 Department of Pathology, Pomeranian Medical University in Szczecin, 1 Unii Lubelskiej St., 71-242 Szczecin, Poland; elzbieta.urasinska@pum.edu.pl; 3 Institute of Biology, University of Szczecin, 13 Wąska St., 71-415 Szczecin, Poland; alicja.trzeciak-ryczek@usz.edu.pl; 4 The Centre for Molecular Biology and Biotechnology, University of Szczecin, 13 Wąska St., 71-415 Szczecin, Poland; 5 Department of Clinical and Molecular Biochemistry, Pomeranian Medical University in Szczecin, 72 Powstańców Wlkp. Al., 70-111 Szczecin, Poland; andrzej.ciechanowicz@pum.edu.pl; 6 Department of Pediatrics, Child Nephrology, Dialysotherapy and Management of Acute Poisoning, Pomeranian Medical University, 4 Maczna St., 70-204 Szczecin, Poland; andrzej.brodkiewicz@pum.edu.pl

**Keywords:** anaerobic effort, CC- and CXC chemokines’ receptors, endurance effort, genes expression, interleukin receptors, LAMPs, leucocytes

## Abstract

This study aimed to assess the post-effort transcriptional changes of selected genes encoding receptors for chemokines and interleukins in young, physically active men to better understand the immunomodulatory effect of physical activity. The participants, aged 16–21 years, performed physical exercise tasks of either a maximal multistage 20 m shuttle-run test (beep test) or a repeated speed ability test. The expression of selected genes encoding receptors for chemokines and interleukins in nucleated peripheral blood cells was determined using RT-qPCR. Aerobic endurance activity was a positive stimulant that induced increased expression of *CCR1* and *CCR2* genes following lactate recovery, while the maximum expression of *CCR5* was found immediately post-effort. The increase in the expression of inflammation-related genes encoding chemokine receptors triggered by aerobic effort strengthens the theory that physical effort induces sterile inflammation. Different profiles of studied chemokine receptor gene expression induced by short-term anaerobic effort suggest that not all types of physical effort activate the same immunological pathways. A significant increase in *IL17RA* gene expression after the beep test confirmed the hypothesis that cells expressing this receptor, including Th17 lymphocyte subsets, can be involved in the creation of an immune response after endurance efforts.

## 1. Introduction

It is commonly accepted that physical activity stimulates inflammation [1,2,3,4,5], which triggers muscle repair and regeneration [6,7,8]. Various forms and intensities of physical effort as the main pro-inflammatory factor have been widely discussed by professional, amateur, and recreational athletes. The previous literature indicates that a significant role in shaping the physical fitness of athletes should be attributed to environmental factors [9,10,11]. Nowadays, it is postulated that lifestyle can induce unique molecular patterns known as life-associated molecular patterns (LAMPs), largely comparable to well-known damage- and pathogen-associated molecular patterns (DAMPs and PAMPs, respectively) [12]. However, they are not sufficient to achieve the sports excellence that is needed to win at the highest level of sports. Wang et al. [13] defined the master level as a “multi-genetic, multifactorial quality determined by the interaction of genes and the environment”. Contemporary theories of sports training more often use tools that allow the study of post-exercise changes, not only at the motoric or physiological but also at the molecular level. Taking the sterile inflammation theory [14,15] into account, one can assume that a properly selected training program evokes the anabolic effect by modulating the immune system.

According to the literature, human cells express at least 20 receptors for at least 46 chemokines, and these are synthesized in large quantities by various cells. Furthermore, many of these receptors are needed to enforce cell migration by allowing the response to concentration gradients of various signals [16,17,18]. Two major groups of chemokines have been identified. First, inflammatory cytokines are synthesized by leukocytes, epithelial and endothelial cells, and fibroblasts (cells related to the immune system) but only as a result of their activation. The second group, called “homeostatic”, is produced without classical activating stimuli. Genes encoding these molecules in humans are grouped into two C-C motif (CC) clusters, two C-X-C motif (CXC) clusters, and many non-clustered or mini-cluster genes [16]. Moreover, there are numerous data that chemokine receptors have been associated with various disorders, including immunodeficiencies, cancer, and inflammatory diseases [18].

However, the interaction of chemokines and their receptors plays a pivotal role in determining the migration pattern and tissue localization of effector T cells during the immune response [19]. Our previous study provided evidence that different types of physical effort trigger a short-term imbalance in the Th1/Th2-related cytokine levels [20,21,22,23], specifically the involvement of different interleukins related to Th1, Th2, and Th17 cell differentiation. Moreover, changes observed in T cell subset distributions after progressive effort [21,22,23] could be manifested in the levels of extracellular DAMP proteins [20]. Local inflammation caused by both endurance and anaerobic effort is initiated in the muscle tissue [1,2] and can influence T-cell differentiation and activation related to extracellular signaling molecules, for example, chemokines and cytokines [5,24].

The negative correlation between the post-effort alterations of selected T cell subsets and the age of participants that was observed among young physically active men [21] is in favor of our postulation that the LAMPs concept should be integrated into an explanation of post-effort immunomodulatory effects. Therefore, this study aimed to assess the post-effort (both aerobic and anaerobic) changes in the transcription of selected genes encoding the receptors for chemokines (*CCR1*, *CCR2*, *CCR3*, *CCR5*, *CXCR1*, *CXCR2*, *CXCR3*, *CXCR4*) and interleukins (*IL2RA*, *IL4R*, *IL6R*, *IL10R*, *IL17RA*, *IFNGR1*, *TNFR1A*, *TNFR1B*) related to leukocyte differentiation, migration, and activation (Table 1). This will allow a better understanding of the immunomodulatory effect of physical effort among young, physically active men.

## 2. Materials and Methods

### 2.1. Participant Demographics

**Figure 1:**
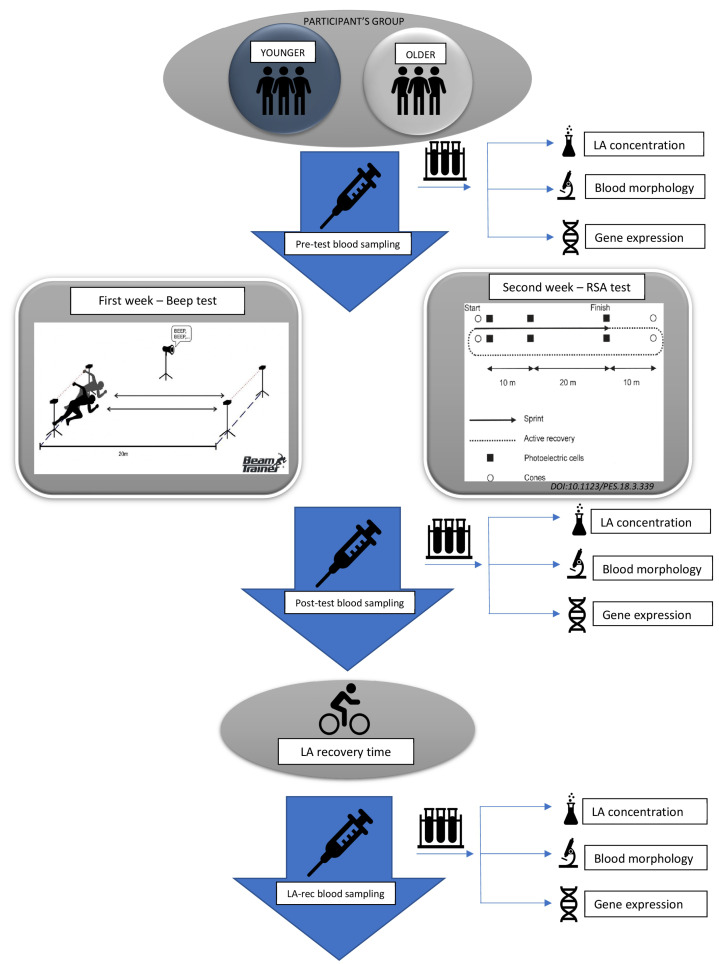
Study design model.

The study was approved by the Local Ethics Committee of the Regional Medical Chamber in Szczecin, Poland (approval no. 05/KB/VII/2019). The study design model is presented in Figure 1.

Forty-two participants aged between 16 and 21 who performed at least 60 min of daily physical activity were recruited to the study. Participants were divided into two age groups according to their age: younger (16–17 years old) and older (19–21 years old), respectively. All participants were non-smokers and refrained from taking any medications or supplements known to affect metabolism. Moreover, they had no history of any metabolic syndrome or cardiovascular diseases. They also did not have medically detected hormonal or immune system disorders.

Participants (or their parents, where appropriate) were provided with the study information, and anyone not meeting the inclusion criteria (e.g., taking medications or supplements affecting metabolism, not giving, or later withdrawing consent) was excluded from the study. Participants were fully informed of any risks and possible discomfort associated with the experimental procedures before giving their written consent to participate.

The body mass, body mass index (BMI), basal metabolic rate (BMR), percentage of fat (FAT), fat mass (FAT MASS), and total body water (TBW) were determined using a Body Composition Analyzer (Tanita BC-418MA, Tokyo, Japan). Cardiorespiratory fitness measures, namely maximal oxygen consumption (VO2max), minute ventilation (VE), anaerobic threshold (AT), respiratory quotient (RQ), respiratory compensation (RC), maximal voluntary ventilation (MVV), metabolic equivalent (MET), and respiratory frequency (Rf), were determined using a state-of-the-art breath-by-breath gas exchange data analyzer Quark CPET (Cosmed, Albano Laziale, Italy) [29] according to a previous protocol [23,30].

### 2.2. The Physical Effort Test

Participants were asked to perform physical exercise tasks according to the protocol, involving (i) a maximal multistage 20 m shuttle-run test (commonly known as a “Beep test”) [31,32] and (ii) a repeated speed ability test (RSA test) [33,34].

Tests were performed indoors (in an athletics hall) at a temperature of 20–23 °C and 2 h after a light breakfast, always beginning with a standardized warm-up of running at a speed of 5 km/h for 10 min. There was a one-week break between the Beep and RSA tests.

### 2.3. Blood Analysis

Blood samples were collected at three time points from the cubital vein, namely before testing (pre-test), no longer than 5–15 min after exercise (post-test), and approximately 1 h later, at the end of the lactate recovery period [35,36,37]. At each time point, venous blood samples were collected in a 7.5 mL S-Monovette tube for serum separation (SARSTEDT AG & Co., Nümbrecht, Germany) and a 7.5 mL S-Monovette tube with ethylenediaminetetraacetic acid (EDTA K3, 1.6 mg EDTA/mL blood) for immune cell analyses (SARSTEDT). All analyses were performed immediately following blood collection and serum separation except for the evaluation of lactate concentration.

It is known that exercise may cause changes in the plasma volume and therefore changes in analyzed cell counts. Therefore, to compensate for this phenomenon, plasma volume loss (Δ*PV*) was calculated according to the classic equations from Dill and Costill, provided by Alis et al. [38]:

$$\Delta PV\left(\%\right)=100\times \left(\frac{Hb_{pre}}{Hb_{post}}\times \frac{100-Htc_{post}}{100-Htc_{pre}}-1\right)$$

where: *Hb_pre_* = hemoglobin pre-test (g/dL); *Hb_post_* = hemoglobin post-test (or in recovery; g/dL); *Htc_pre_* = hematocrit pre-test (%); and *Htc_post_* = hematocrit post-test (or in recovery; %).

Then, the corrected blood parameter values were calculated using the formula:

$$\left[Corrected\;parameter\;concentration\right]=\left[Uncorrected\;parameter\;concentration\right]\times \left(1+\frac{\Delta PV\left(\%\right)}{100}\right)$$

#### 2.3.1. Determination of Lactate Level

The serum was separated from whole blood by centrifugation of blood collecting tubes at 2000× *g* for 10 min at room temperature. Following serum collection, the lactate concentration was determined with a colorimetric assay kit (PZ Cormay S.A., Łomianki, Poland) according to the manufacturer’s protocol using an Automatic Clinical Chemistry BM-100 Analyzer (BioMaxima S.A., Lublin, Poland). To confirm lactate recovery, the point when lactic acid (LA) concentration returned to the pre-exercise level, LA concentration was determined at three time points: pre-test, post-test, and 1 h post-effort (LA-rec).

#### 2.3.2. Hematological Analysis

The hematological analysis was performed immediately after the blood collection. Hematocrit, hemoglobin concentration, and white blood cell (WBC) count, including lymphocyte (LYM), granulocyte (GRA), and monocyte (MON) counts, were analyzed using a hematology analyzer ABX Micros 60 (Horiba ABX, Warsaw, Poland).

#### 2.3.3. Total RNA Isolation

Total RNA was isolated from the peripheral blood leukocyte pellet using the GeneMatrix Universal RNA Purification Kit (EURx, Gdańsk, Poland) according to the manufacturer’s protocol. The red blood cells were lysed before RNA isolation according to the manufacturer’s protocol. RNA samples were purified of any remaining DNA by treatment with DNase I enzyme (EURx). The RNA concentration and purity of each sample were evaluated using a NanoDrop™ 2000 Spectrophotometer (Thermo Fisher Scientific, Waltham, MA, USA).

#### 2.3.4. Gene Expression Determination

First-strand cDNA of each sample was synthesized from 2 µg of DNase-treated total RNA in a 20 µL total reaction volume, using the RevertAid RT Kit (ThermoFisher Scientific) according to the manufacturer’s protocol. cDNA samples were diluted 10x with nuclease-free water and stored at −20 °C for further analysis.

Amplification of selected genes from a cDNA template was performed by qPCR using the PowerUp^TM^ SYBR^®^ Green Master Mix (Applied Biosystems^TM^, ThermoFisher Scientific) on a CFX96 Touch Real-Time PCR Detection System (Bio-Rad, Hercules, CA, USA). The primers used in the qPCR analyses are listed in Table 2. Cycling conditions (temperature and time) were determined according to the manufacturer’s instructions, taking into consideration the melting temperatures of primers and the length of expected amplicons. Additionally, to exclude nonspecific products, the melting curves of PCR products were analyzed after the termination of the reaction. The reaction products obtained from each pair of primers were sequenced to confirm the results (Supplementary Figures S1–S19).

Amplification reactions were validated so that the efficiency was more than 95% for all the tested genes. Therefore, Livak’s comparative method (*∆∆Ct*) [39] was used to calculate the fold-change in gene expression, normalized to an average of three reference genes, namely *ACTB*, *B2M*, and *RACK1*, relative to pre-test control results (post-test/pre-test ratio and LA-rec/pre-test ratio, respectively). Each sample was analyzed in triplicate to increase the precision of the data.

### 2.4. Statistical Analysis

All data are presented as median values (interquartile range), except for age, which is presented as median (minimum–maximum range). Non-parametric statistical tests were used as the data distribution was verified to be non-normal using a Shapiro–Wilk test. The significance level of differences observed between time points (pre-exercise versus post-exercise versus lactate recovery) was calculated using Friedman’s analysis of variance for repeated measures, followed by Dunn’s post hoc test with Bonferroni correction. The significance level of differences in parameters between the Beep and RSA tests or between younger and older groups was calculated using the Mann–Whitney U-test. The correlations between analyzed parameters were assessed using Spearman’s rank correlation coefficient determination. A *p*-value of <0.05 was considered significant. Statistical analysis was performed using Statistica v.13 software (2017; TIBCO Software Inc., Palo Alto, CA, USA).

## 3. Results

Our participants performed two types of effort, namely aerobic (Beep test) and anaerobic (RSA test) (Figure 1). Both age groups were comparable in their cardiorespiratory characteristics and body composition, confirming the homogeneity of the whole cohort. Only age and length of training experience differed significantly between the younger and older groups (Table 3).

Unfortunately, not all participants were able to take part in the second anaerobic test, hence the older group has a smaller number of participants for the RSA than for the Beep test. The results of the performed physical efforts tests are presented in Table 4. Both age groups were homogenous and there were no significant differences between the results of both performed aerobic and anaerobic tests.

To confirm the efficiency of lactate recovery, the corrected LA concentrations were analyzed (Table 5). The statistical analysis demonstrated no significant differences between age groups for LA concentration following the two types of physical effort, and the LA values found 60 min after cessation of either the Beep or RSA tests, were comparable to baseline (pre-test) LA concentration values. The corrected LA concentration found as post-effort values in each studied group after the Beep and RSA tests, respectively, are related to the biogenetic characteristics of those types of effort [31,32,33,34].

It was found that the Beep test caused significant increases in corrected WBC, LYM, MON, and GRA counts at the post-test time point in comparison to baseline values among all participants as an immediate post-effort cellular response. Corrected LYM counts at the LA-rec time point following the Beep test were significantly lower, while corrected GRA counts were significantly higher than baseline values in both age groups. The increase in corrected WBC counts was also noticed in the LA-rec time point in comparison to pre-test values. Post-effort increases in corrected WBC, MON, and GRA counts after the RSA test were observed in both age groups in comparison to baseline values. However, no significant change in corrected LYM count was found after the RSA test in the older group. The increase in corrected GRA counts in comparison to the pre-test time point was noticed only during the LA-rec after the RSA test in both age groups (Table 6).

Based on the Mann–Whitney U test analysis, there were no significant differences in corrected WBC, LYM, MON, and GRA counts after physical effort (both aerobic and anaerobic) and LA recovery between the studied age groups. Conversely, significantly higher corrected GRA counts were found in the post-test time point, although only in the younger group after the RSA test, and compared to the Beep test (pMW = 0.0327).

**Figure 2:**
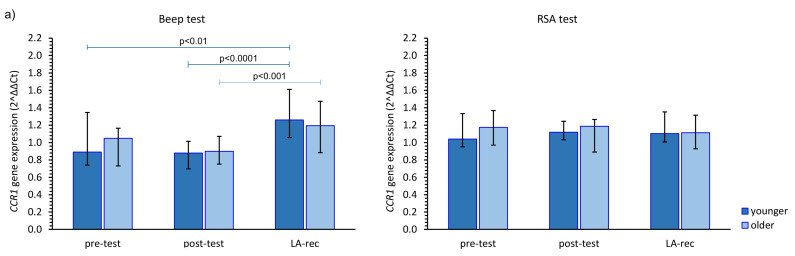

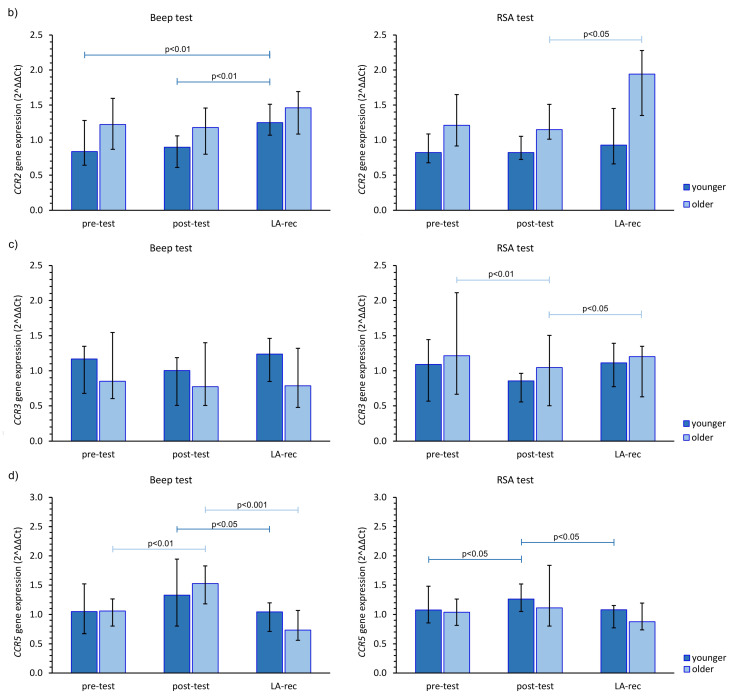
The impact of aerobic (Beep test) and anaerobic (RSA test) effort on the relative expression of studied genes encoding (**a**) *CCR1*, (**b**) *CCR2*, (**c**) *CCR3*, (**d**) *CCR5*. The figures present median (interquartile range) values. Significance levels of differences observed between analyzed time points (pre-test vs. post-test vs. LA-rec) were assessed using Friedman’s analysis of variance for repeated measures followed by post hoc Dunn’s test with Bonferroni correction.

The impact of the studied physical effort types on *CCR* gene expression in both age groups was heterogeneous. The aerobic effort caused an increase in *CCR1* gene expression, but only after LA-rec in comparison to baseline values, regardless of the age group, while no significant differences were observed after the anaerobic effort (Figure 2a). Expression of the *CCR2* gene was significantly higher after the Beep test in both studied time points when compared to the pre-test values among the younger group, and only after the LA-rec time point following the RSA test in the older group of the participants (Figure 2b). We found significant differences in the expression of the *CCR2* gene observed after the RSA test in both post-test and LA-rec time points between younger and older groups (pMW = 0.0058 and pMW = 0.0036, respectively). It was found that in the older group, the post-test expression of the *CCR3* gene was significantly lower than both baseline and LA-rec values only after the RSA test (Figure 2c). The aerobic effort during the Beep test induced a decrease in *CCR5* gene expression but only following the LA-rec time point in comparison to post-effort values in the younger group. Conversely, the same physical effort in the older group triggered an approximately 1.4-fold increase in *CCR5* gene expression as an immediate post-effort response. Likewise, a post-effort change in *CCR5* expression was found following the RSA test in the younger group (Figure 2d).

**Figure 3:**
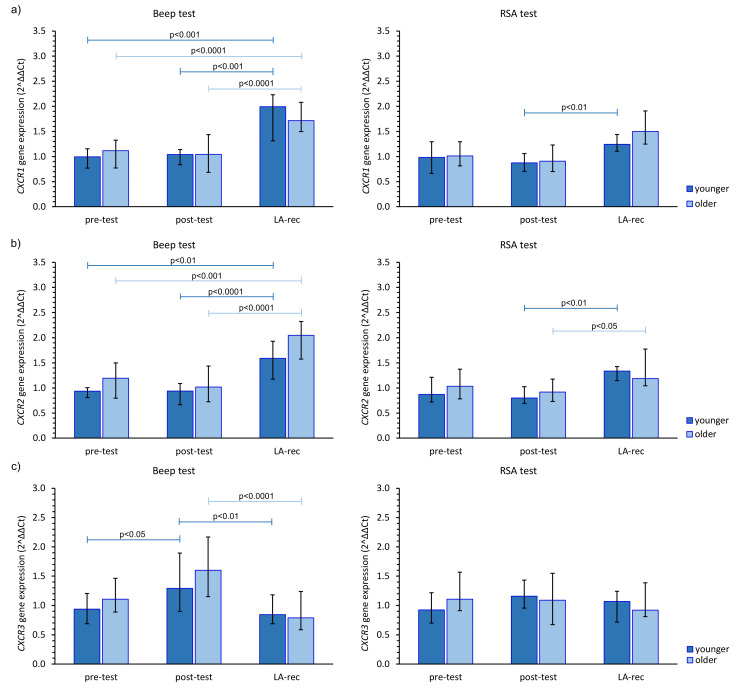

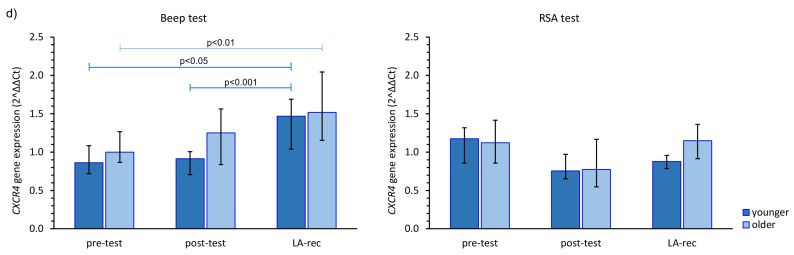
The impact of aerobic (Beep test) and anaerobic (RSA test) effort on the relative expression of studied genes encoding (**a**) *CXCR1*, (**b**) *CXCR2*, (**c**) *CXCR3*, (**d**) *CXCR4*. The figures present median (interquartile range) values. Significance levels of differences observed between analyzed time points (pre-test vs. post-test vs. LA-rec) were assessed using Friedman’s analysis of variance for repeated measures followed by post hoc Dunn’s test with Bonferroni correction.

The immune response to the physical efforts was demonstrated via *CXCR* gene expression. After completing the Beep test, there was an increase in the expression of these genes at the LA-rec time point, regardless of age group, while after the RSA test, no statistically significant alterations in those genes’ expression were observed, except for *CXCR1* gene in the younger group and *CXCR2* gene in both age groups (Figure 3). The aerobic effort caused an approximately 1.5–2.0-fold increase in the expression of *CXCR1*, *CXCR2*, and *CXCR4* gene expression during LA-rec in comparison to baseline values in both age groups (Figure 3a,b,d). Furthermore, there was a significant (pMW = 0.0294) difference in the expression of *CXCR2* observed after the Beep test at the LA-rec time point between the younger and older group. Taking the *CXCR3* gene expression into account, a significant decrease was observed at the LA-rec time point in comparison to the post-test time point after completion of the Beep test (Figure 3c). No significant change in any *CXCR* genes was found after the RSA test in either age group (Figure 3). However, a significant (pMW = 0.0314) difference in the expression of *CXCR4* gene was observed between the young and old groups following the RSA test at the LA-rec time point.

**Figure 4:**
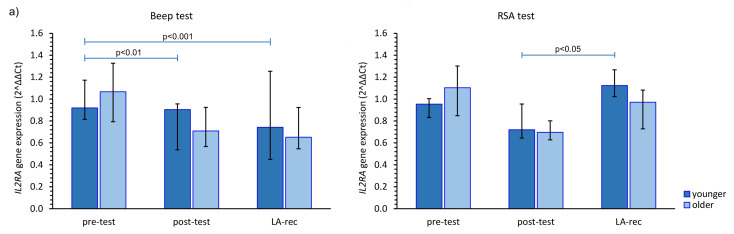

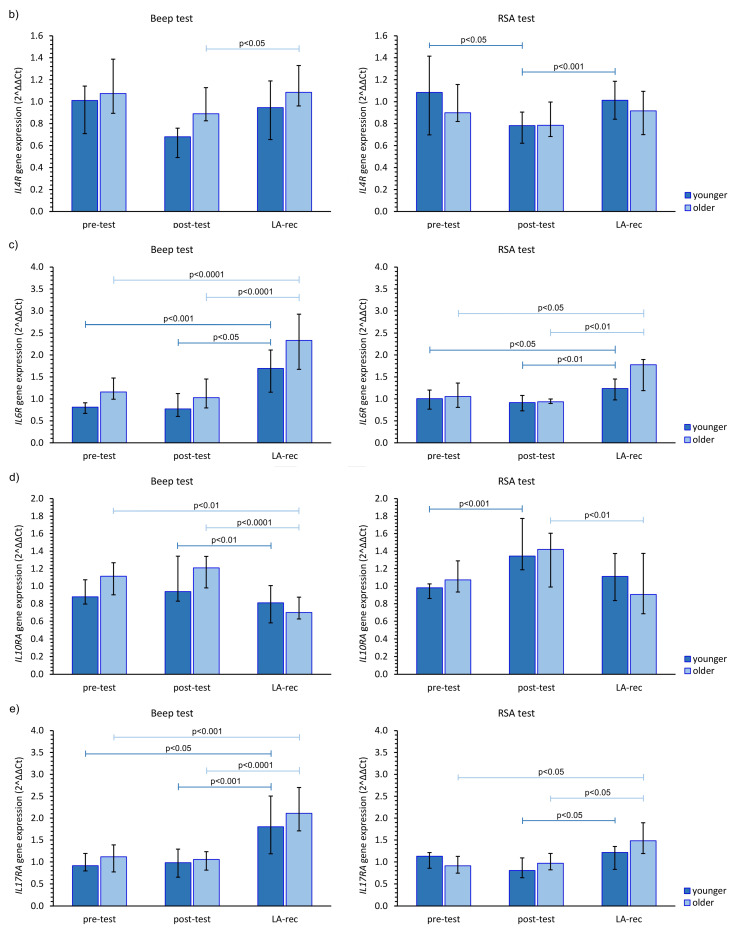
The impact of aerobic (Beep test) and anaerobic (RSA test) effort on the relative expression of studied genes encoding (**a**) *IL2RA*, (**b**) *IL4R*, (**c**) *IL6R*, (**d**) *IL10RA*, (**e**) *IL17RA*. The figures present median (interquartile range) values. Significance levels of differences observed between analyzed time points (pre-test vs. post-test vs. LA-rec) were assessed using Friedman’s analysis of variance for repeated measures followed by post hoc Dunn’s test with Bonferroni correction.

We found that the aerobic effort did not cause an increase in *IL2RA* gene expression in either age group. However, in the younger group, the increase in the expression of this gene was found at the LA-rec time point following completion of the RSA test in comparison to baseline values (Figure 4a). No significant increase compared to pre-test values in *IL4R* gene expression was observed in our study (Figure 4b). Furthermore, we found a significant (pMW = 0.0023) difference in the *IL4R* gene expression following the Beep test at the post-test time point between the younger and older groups. Conversely, regardless of the effort or age of the participants, a significant increase in *IL6R* was found at the LA-rec time point, pointing to a lactate recovery effect of the immune system (Figure 4c). Moreover, we found significant differences in the expression of *IL6R* at the LA-rec time point between age groups following both the aerobic and anaerobic efforts (pMW = 0.0112, pMW = 0.0314 for Beep and RSA tests, respectively). A significant increase in *IL10RA* gene expression in the young participant group was found as an immediate post-RSA response, while a significant decrease in *IL10RA* expression was found at the LA-rec time point in comparison to all other time points in the older group of participants (Figure 4d). Excluding the young RSA group, a significant increase in *IL17RA* was found as a lactate recovery effect of immune response (Figure 4e), with significant (pMW = 0.0285) differences in the expression of *IL17RA* observed following completion of the RSA test at the LA-rec time point between the younger and older groups.

**Figure 5:**
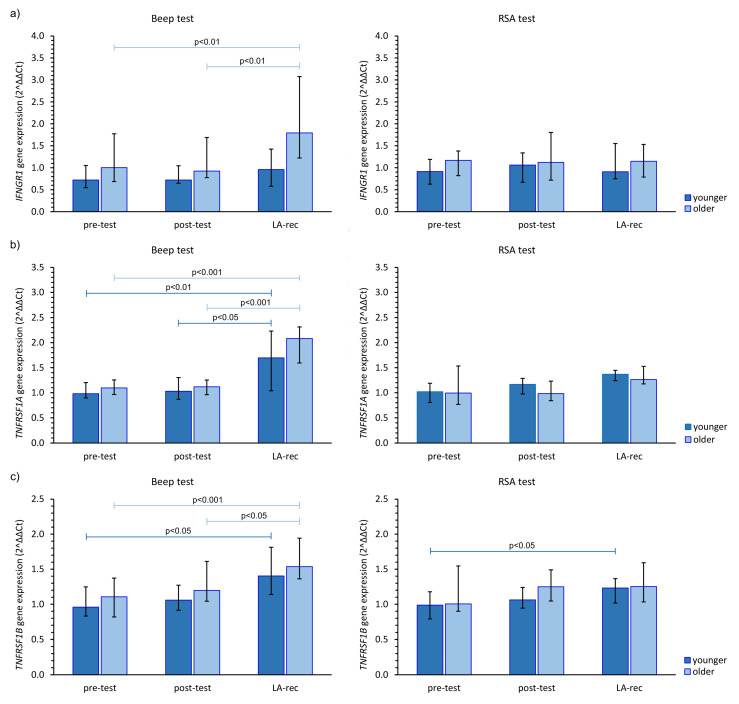
The impact of aerobic (Beep test) and anaerobic (RSA test) effort on the relative expression of studied genes encoding (**a**) *IFNGR1*, (**b**) *TNFRSF1A*, (**c**) *TNFRSF1B*. The figures present median (interquartile range) values. Significance levels of differences observed between analyzed time points (pre-test vs. post-test vs. LA-rec) were assessed using Friedman’s analysis of variance for repeated measures followed by post hoc Dunn’s test with Bonferroni correction.

Finally, we found the Beep test triggered an increase in *IFNGR1* expression at the LA-rec time point in comparison to baseline values in the older group of participants (Figure 5a). Interestingly, anaerobic exercise did not cause a significant change in *IFNGR1* gene expression regardless of age group. Significant (pMW = 0.0029) differences in the expression of *INFGR1* were observed after the Beep test at the LA-rec time point between the younger and older groups. As a person’s lactate recovery affects their Beep test results, significant increases in *TNFR1A* and *TNFR1B* expression were found in both age groups (Figure 5b,c). Interestingly, significant increases in both *TNFR1* genes were only found in young participants at this time point following the RSA test (Figure 5b,c).

To verify the effect of age on the response to exercise, correlation coefficients between participants’ age and corrected LA concentration, number of analyzed cells or analyzed chemokine and cytokine gene expression at the studied time points were calculated and are provided in the supplementary Table S1. Statistically significant correlations are presented in supplementary Figures S20 and S21. The correlation of expression of chemokine and cytokine receptors with the number of peripheral blood cells was also verified. In the younger group performing the Beep test, we observed significant correlations between corrected WBC count and *CXCR4* gene expression (R = 0.60; *p* = 0.0109) at the pre-test time point, between corrected WBC count and *IL10R* (R = −0.52; *p* = 0.0316) and *TNFR1B* (R = −0.48; *p* = 0.0483) gene expression at the post-test time point. Regarding older participants performing the Beep test, WBC count correlated only with *IL17RA* gene expression in post-test (R = −0.46; *p* = 0.0191). In younger participants performing the RSA test, a negative correlation between corrected WBC count and *TNFR1B* gene expression (R = −0.50; *p* = 0.0410) in LA-rec was observed. In the case of older athletes, WBC count correlated with *IL2RA* gene expression (R = 0.54; *p* = 0.0365; pre-test), *IL17RA* gene expression (R = −0.71; *p* = 0.0028; post-test), and *CXCR3* and *CXCR4* gene expression (R = 0.58; *p* = 0.0238 and R = 0.51; *p* = 0.0498, respectively; LA-rec time point).

## 4. Discussion

The results of this study demonstrated that both the age of the participants and the type of effort can have a significant impact on the expression of selected genes that encode receptors for chemokines and cytokines related to leukocyte migration, differentiation, and activation. It was found that, regardless of the type of effort, an increase in WBC, LYM, and MON absolute counts was an immediate post-effort effect noticed in peripheral blood, and this effect did not persist at the end of LA-rec. However, increases in GRA absolute counts were observed at this time, and this result is in line with the previous literature data [21,40,41,42]. We have previously shown that a change in selected T cell subsets is only present at the post-test time point, suggesting that the older the participant is, the weaker the biological effect of the effort would be [21]. Moreover, as a short-term biological effect of aerobic efforts, the involvement of Th17 cells in the post-effort immune response and its probable role in differentiation into Treg cells has been described [20,21,22]. Additionally, our previous study on endurance efforts indicated that mechanisms related to the involvement of Th1 and Th2 cell subsets in the post-effort response appear to be related to a participant’s age [21]. Conversely, an increase in Th2 cell-related cytokines was found, yet without similar changes in the cell distribution [21], and the lack of increases in the level of phosphorylated JAK/STAT or Ras/MAPK proteins as a post-effort effect among young physically active men [43]. This suggests that those observations can be related to the recruitment of activated lymphocytes that are circulating in peripheral blood. From this point of view, the LAMPs hypothesis seems to be logical and aids in the explanation of this finding, especially since the present results are in line with previous hypotheses and observations. It should be pointed out that in our previous studies [21,23] we used no [23] or different [21] age group stratification. However, these previous studies could be treated as preliminary research leading to the division into age groups presented in this study.

### 4.1. The Impact of Aerobic and Anaerobic Effort on the Expression of Genes Encoding Selected Chemokines Receptors

It is well known that activated T cells clonally expand and differentiate in the secondary lymphoid organs and then migrate to the source of the antigens [18,44] excluding *CCR1* gene expressed mainly by granulocyte and monocyte subsets. The expression of other studied genes was observed in different subsets of lymphocytes [26,27,28]. The results of this study demonstrate that the leukocyte *CCR* gene expression profile is altered following aerobic efforts in favor of high *CCR1*, *CCR2*, and *CCR5* gene expression, yet no change in *CCR3* expression was observed. Aerobic endurance effort was a positive stimulant that induced high expression of *CCR1* and *CCR2* genes at the LA-rec time point, while the alteration in *CCR5* gene expression was found as an immediate post-effort response. These findings are in line with the increasing absolute counts of LYM and MON found in this study. One possible explanation of the increasing expression of *CCR1* and *CCR2* genes during the LA-rec time point could be the increase in GRA absolute count observed at this time point in both studied groups. In contrast to this observation, the anaerobic effort was not a strong enough stimulant to induce changes in the expressions of *CCR* genes in the younger group of participants. The increase in absolute counts of WBC, GRA, LYM, and MON found in both studied age groups did not correlate with the increased expression of studied *CCR* genes. Previous work has found that an increase in *CCR1*, *CCR2*, *CCR3*, and *CCR5* gene expression was temporally correlated with meningeal inflammation and required the presence of functional T cells, while it was not necessary to induce IFN-γ [19]. The results of our study seem to demonstrate that endurance efforts can be treated as a sterile inflammatory factor. The chemokine-related regulation is particularly important for inducible chemokine receptors, such as CCR2 and CCR5 helping to recruit blood neutrophils, monocytes, and activated T cells to the sites of infection [45,46]. The changes in gene expression and cell counts observed in the present and previous studies [21,22,23] are in line with this phenomenon. However, this pattern of changes is different following an anaerobic effort, as only *CCR2* genes from leukocytes are involved in the recovery response among participants with longer training experience.

Previous investigations have found that reactive oxygen intermediaries, for example those produced by phagocytes to kill pathogens, increased *CCR2*, *CCR5*, and *CXCR4* mRNA expression [18,47]. The changes induced by the physical efforts undertaken in this study can also be related to oxidative stress triggered by this physiological response. Similar to the observed *CCR* expression changes, aerobic efforts seem to be a stronger stimulant than anaerobic efforts in increasing the expression of *CXCR1*, *CXCR2*, and *CXCR4* genes. Only the expression of the *CXCR3* gene was not increased by aerobic efforts among older, physically active men. According to literature data, the *CXCR3* gene is expressed mainly in LYM subtests, including T and NKT cells [26,27]. In this study, a significant post-effort increase in LYM absolute count was found in both studied groups. Taking the T lymphopoietic role of CXCR3 [48] into account, it would explain the increases in naïve T cells as the late immunological effect of the endurance efforts observed in young men that have been described earlier [21,22,23]. Conversely, the increase in *CXCR4* gene expression can also help to explain not only the observed LA-rec increase in T cell subsets in peripheral blood [21,22,23] because of its hematopoietic role [48] but also the increase in IL-8 secretion, which has been described as one of the biological effects of endurance efforts [21,22,23].

Neutrophils conventionally express CXC chemokine receptors, while CC chemokine receptors are generally absent, making these cells unresponsive to stimulation by CC chemokine ligands [44,48]. This may explain the increase in GRA counts and increase in *CXCR* gene expression during the recovery phase following aerobic efforts. On the molecular level, chemokine receptor expression, for example *CXCR1* and *CXCR2* on activated neutrophils or *CCR2* during monocyte differentiation, has been described as an efficient method to down-regulate the mechanisms related to protein degradation independently of, or in combination with, a transcriptional control [18].

The cooperation of one of the chemokine-chemokine receptor pairs, namely monocyte chemotactic protein (MCP)-1/CCR2, is required for monocytes to infiltrate the injured vessel wall and then trigger the proliferation of smooth muscle cells [49]. On the other hand, the pair of stromal cell-derived factor (SDF)-1 alpha/CXCR4 takes part in the neointimal enrollment of smooth muscle progenitor cells as a result of myocyte apoptosis [49]. The activated T lymphocytes expressing CCR1 and CCR5 are involved in chemoattraction of monocytes and neointimal growth [49]. One of the possible explanations of the post-effort anabolic effect observed after the endurance aerobic effort can be related to the *CCR1*, *CCR2,* and *CXCR4* genes expression found in this study.

The role of CCR2 is related to the chemotactic response of monocytes. Moreover, the modulation of CCR2 expression can be positively stimulated by IL-2 and IL-10, and negatively stimulated by IFN-γ, TNF-α, and IL-1 [50]. On the other hand, the study on the mice model evidenced that expression of CCR2 was a major mediator of macrophage recruitment and transport and host defense against bacterial infection [51,52,53]. The chemokine ligand MCP-1 is a potent *in vitro* monocyte activator that is abundantly expressed in a range of pathological conditions characterized by monocyte infiltration [54]. It has been evidenced that differentiation of monocytes into macrophages results in a significant increase in the number of cells that express CCR5 with parallel, a progressive decrease in the expression of CXCR4 at the plasma membrane of those subsets [55]. These data help to explain the increase in the MON subset as an immediate post-effort effect noticed in participants belonging to all studied age groups, regardless of the type of endurance effort.

The increase in expression of inflammation-related genes [48] encoding chemokine receptors (*CCR1*, *CCR2*, *CCR3*, *CXCR1*, *CXCR2*, *CXCR3*) induced by aerobic endurance efforts strengthens the theory that physical effort belongs to the factors that induce sterile inflammation. The results of this study are in line with the pleiotropic observations of Radom-Aizik et al. who observed e.g., alteration in both pro- and antiapoptotic genes and genes being responsible for inflammation, growth, and repair in neutrophils after brief and heavy exercise [56]. Additionally, the different chemokine receptor expression profiles induced by short-term anaerobic effort suggest that not every type of physical effort will activate similar immunological pathways.

### 4.2. The Impact of Aerobic and Anaerobic Effort on the Expression of Genes Encoding Selected Interleukins Receptors

Post-effort secretion of pro- and anti-inflammatory interleukins is well described in the previous literature [57,58,59,60]. Indeed, our previous study provided evidence that endurance efforts triggered a significant increase in pro-inflammatory IL-6 and IFN-γ levels. However, at the same time, comparable levels of anti-inflammatory IL-4 and IL-10 were observed [20]. *IL2RA* gene is expressed in lymphocytes, including T and NK cells, while *IL4R* gene is also expressed in basophiles [25]. *IL6R*, *IL10RA*, *IFNGR1*, and *TNFSRFs* genes are expressed in nearly all WBC subsets in opposition to the *IL17RA* gene being expressed in MON (monocytes and macrophages) and LYM subsets [25]. It was found in this study that an aerobic endurance effort caused an increase in the expression of genes encoding *IL6R*, *IFNGR1*, and both *TNFRSR1*s combined with the post-effort increase in WBC, LYM, and MON absolute counts. Furthermore, we observed a lack of increase in *IL2RA* and *IL10RA* gene expression, and the patterns of *IFNGR1* expression were different depending on the age group regardless of the post-effort increase in LYM absolute count. The increase in *IL6R* expression seems to be in line with previous observations [21,22,23], showing the stimulation of IL-6 secretion as part of a post-effort immune response. IL-6 is responsible for the co-activation of T cells and is associated with their proliferation. Moreover, IL-6 does not inhibit IL-2 production [61], but in the present study, it was found that *IL2RA* gene expression was decreased in young participants.

We found a significant increase in *IL17RA* gene expression combined with an increase in LYM absolute count in both age groups following the Beep test, which seems to confirm the hypothesis that cells expressing this receptor, including Th17 lymphocytes, may be involved in the activation of the immune system as a response to the endurance effort. The participation of Th17 cells in the post-effort immune response in physically active young men was also observed in our previous studies [21,22]. Furthermore, the literature states that the promotion of Th17 cell differentiation is related to an increase in IL-6 [62,63], and our results correspond with this data. Interestingly, in the case of *IL17RA* and *IL6R* gene expression, both aerobic and anaerobic efforts induced a similar response. This mechanism is possibly independent of the type of effort.

Our previous study indicated that an aerobic effort is not enough stimulus to activate the phosphorylation of JAK/STAT and Ras/MAPK signaling pathway-related proteins (ERK1/2, p38 MAPK, STAT1, STAT3, STAT5, and STAT6) in T cells [43]. The results of this study are in favor of the hypothesis that the lactate recovery period (up to 1 h after completing an effort) may not be enough time to induce T cell activation and differentiation. The results of the present experiment provide evidence that following lactate recovery, molecular mechanisms are being activated that influence the expression of genes related to the activation and differentiation of leukocytes.

## 5. Conclusions

The mechanisms responsible for the creation and activation of immunological pathways are triggered by different external and internal factors and involve a different subset of cellular components, including T cells. These mechanisms need to be modulated by chemical intermediaries, namely chemokines and interleukins. Cellular traffic during an inflammatory response induced by sterile inflammatory factors occurs in a similar spatial and temporal fashion to other inflammatory inducers. The resulting inflammatory process leads to an alteration in the immune balance, which results in para- and autocrine changes facilitating the release and activation of signaling factors. At the molecular level, it induces changes in the expression of genes encoding not only signaling molecules but also their receptors. Even though physical effort can induce an increase in the expression of genes responsible for inflammation in nucleated cells and both pro- and anti-apoptotic-related genes at the same time, it is an important external signal for the recruitment of the immune system to respond to future threats. All observed changes in gene expression studied herein seem to be a balanced response related to the age of the participants, the type of physical effort, and the duration of the exercise. The late immunological consequences of these factors, and the influence of subsequent physiological stimuli, lead to changes in the cellular components of blood and immune system function.

## Figures and Tables

**Table 1:** White blood cells expressing analyzed genes encoding receptors for chemokines and interleukins [25,26,27,28].

Gene	White Blood Cells Expressing Given Gene
*CCR1*	GRA: neutrophils, eosinophils, basophils; MON: monocytes
*CCR2*	GRA: basophils; LYM: T cells, NK cells
*CCR3*	GRA: eosinophils, basophils; LYM: T cells
*CCR5*	LYM: Th cells; MON: monocytes
*CXCR1*	GRA: neutrophils, eosinophils, basophils; LYM: T cells, NK cells; MON: monocytes
*CXCR2*	GRA: neutrophils; LYM: NK cells; MON: monocytes
*CXCR3*	LYM: T cells, NKT cells
*CXCR4*	GRA: neutrophils, eosinophils, basophils; LYM: all subsets; MON: all subsets
*IL2RA*	LYM: T cells, NK cells
*IL4R*	GRA: basophils; LYM: T cells
*IL6R*	GRA: neutrophils, eosinophils, basophils; LYM: all subsets; MON: all subsets
*IL10RA*	GRA: neutrophils, eosinophils, basophils; LYM: all subsets; MON: all subsets; DC
*IL17RA*	LYM: T cells, B cells; MON: monocytes, macrophages
*IFNGR1*	GRA: neutrophils; LYM: T cells, NK cells; MON: monocytes
*TNFRSF1A*	GRA: neutrophils
*TNFSR1B*	MON: monocytes

DC—dendritic cells, GRA—granulocytes, LYM—lymphocytes, MON—monocytes, NK—natural killer cells, Th—T helper cells.

**Table 2:** qPCR primers used in this study.

Gene	Forward Primer	Reverse Primer	Amplicon Length (bp)	T_M_ of the Amplification Products (°C) ^1^
*CCR1*	CCCTTGGAACCAGAGAGAAGCC	CAAAGGCCCTCTCGTTCACC	122	82.5
*CCR2*	AGCCACAAGCTGAACAGAGA	TGGTGACTTCTTCACCGCTC	127	80.5
*CCR3*	GGAGAAGTGAAATGACAACCT	TTTTCACAGAGCAGGCCCAC	89	78
*CCR5*	AACTCTCCCCGGGTGGAAC	ACCAGTGAGTAGAGCGGAGG	141	81
*CXCR1*	TGGCCGGTGCTTCAGTTAGA	AGGGGCTGTAATCTTCATCTGC	136	79.5
*CXCR2*	GCTCTTCTGGAGGTGTCCTAC	TAGTAGAAAAGGGGGCAGGGTA	170	80.5
*CXCR3*	TGCTAAATGACGCCGAGGTT	GGAGGTACAGCACGAGTCAC	92	81
*CXCR4*	TGGTCTATGTTGGCGTCTGG	GTCATTGGGGTAGAAGCGGT	116	81.5
*IL2RA*	GCAGAGAAAGACCTCCGCTT	TGATGAACGTGAGCAGTCCC	95	82.5
*IL4R*	AGCGTTTCCTGCATTGTCATC	AATTCTTCCAGTGTGGGCACTT	202	85.5
*IL6R*	TCAGTGTCACCTGGCAAGAC	GGAGGTCCTTGACCATCCAT	120	81.5
*IL10RA*	CTCCTCAGCCTCCGTCTTGG	GGGTCTGGCTACAGTTGGAGA	202	84
*IL17RA*	ACGTTTGTGCGTCAGGTTTG	TTGGACTGGTGGTTTGGGTC	165	84.5
*IFNGR1*	TGCTGTATGCCGAGATGGAAA	ATCGACTTCCTGCTCGTCTC	124	77.5
*TNFRSF1A*	TCCAAATGCCGAAAGGAAATG	ACACGGTGTTCTGTTTCTCCT	190	84
*TNFRSF1B*	GCATTTACACCCTACGCCCC	GAGTTTCCACCTGGTCAGAGC	241	86.5
*ACTB*	CATGTACGTTGCTATCCAGGC	CTCCTTAATGTCACGCACGAT	250	88
*B2M*	GAGGCTATCCAGCGTACTCCA	CGGCAGGCATACTCATCTTTT	248	80
*RACK1*	GAGTGTGGCCTTCTCCTCTG	GCTTGCAGTTAGCCAGGTTC	224	84.5

^1^ T_M_—melting temperature.

**Table 3:** The characteristic of the participants.

	Younger Group (*n* = 17)	Older Group (*n* = 25)	p_MW_ ^1^
Age (years)	17 (16–17)	20 (19–21)	0.0000
Height (cm)	182 (178–187)	181 (179–186)	0.9177
Weight (kg)	73.9 (71.0–79.3)	76.1 (70.2–83.2)	0.3155
BMI (kg/m^2^)	22.3 (21.0–23.8)	23.3 (21.6–24.4)	0.1116
BMR (kJ)	8632 (7966–9037)	8447 (7914–8855)	0.2348
Fat (%)	11.5 (7.9–12.3)	11.2 (8.2–13.2)	0.8494
Fat mass (kg)	8.2 (5.6–9.9)	8.6 (5.4–10.9)	0.6920
FFM (kg)	66.6 (62.3–68.5)	68.3 (64.5–70.9)	0.2101
TBW (kg)	48.8 (45.6–50.1)	50.0 (47.2–51.9)	0.2023
VO_2_max (mL/kg/min)	60.9 (58.4–65.0)	60.7 (57.8–65.9)	0.7819
VE (L/min)	151 (143–157)	143 (125–157)	0.3534
RQ	1.07 (1.04–1.08)	1.07 (1.05–1.09)	0.0771
AT (beats/min)	165 (154–176)	166 (159–182)	0.1501
RC	178 (170–189)	184 (176–193)	0.0579
MVV (L/min)	190 (182–200)	185 (175–194)	0.1194
MET (mL/kg/min)	17.4 (16.7–18.1)	18.2 (17.0–18.9)	0.0566
Rf	64.6 (56.8–68.3)	60.3 (56.8–65.5)	0.1568
HRmax (beats/min)	201 (191–208)	198 (194–202)	0.8319
Training experience (years)	9 (9–11)	12 (10–14)	0.0000
Weekly training volume (h)	10.0 (10.0–10.5)	11.0 (10.0–12.5)	0.0463

The table presents the median (interquartile range), except for age, which is presented as median (minimum–maximum range) values characterizing the participants. BMI—body mass index, BMR—basal metabolic rate, FFM—fat-free mass, TBW—total body water, VO_2_max—maximal oxygen uptake, VE—minute ventilation, RQ—respiratory quotient (volume ratio of emitted CO_2_ to oxygen uptake), AT—anaerobic threshold, RC—respiratory compensation, MVV—maximal voluntary ventilation, MET—metabolic equivalent, Rf—respiratory frequency, HRmax—maximum heart rate, *n*—number of participants. ^1^ Differences between groups (younger vs. older group) were assessed using the Mann–Whitney U-test.

**Table 4:** The results of the tests performed by the participants.

	Younger Group (*n* = 17)	Older Group (*n* = 25)	p_MW_ ^1^
Beep decimal score	13.2 (11.3–14.5)	13.7 (11.5–15.2)	0.3417
RSA mean score (s)	2.68 (2.15–2.92)	2.82 (2.13–3.26)	0.3766

The table presents median (interquartile range) values characterizing the participants’ test performances. ^1^ Differences observed between analyzed groups (younger vs. older group) were assessed using the Mann–Whitney U test. Beep—maximal multistage 20-m shuttle run test, RSA—repeated speed ability test.

**Table 5:** The corrected lactate concentration (mmol/L) in the participants’ blood.

	Younger Group		Older Group	
	Beep Test	RSA Test	Beep Test	RSA Test
pF ^1^	0.0000	0.0000	0.0000	0.0006
pre-test	2.2 (1.9–2.3) ^aaaa^	3.2 (3.1–3.6) ^aa^	2.0 (1.8–2.4) ^aaaa^	3.2 (3.0–3.4) ^a^
post-test	7.7 (7.0–8.8) ^bbbb^	14.9 (12.5–16.0) ^bbbb^	8.1 (7.3–8.7) ^bbbb^	14.12 (10.5–16.1) ^bbb^
LA-rec	2.1 (1.8–2.3)	3.0 (2.8–3.1)	2.3 (2.1–2.8)	2.9 (2.8–3.2)

^1^ Significance levels of differences observed between time points (pre-test vs. post-test vs. recovery) were assessed using Friedman’s analysis of variance for repeated measures (pF—Friedman’s ANOVA *p*-values) followed by Dunn’s post hoc test with Bonferroni correction. The table presents the median (interquartile range) of values corrected for *ΔPV*. Beep—maximal multistage 20-m shuttle run test, RSA—repeated speed ability test. The analyses were performed before (baseline, pre-test) and after the effort (approximately 5 min post-effort and during LA-rec approximately 1 h after the test). Post-hoc *p*-values: ^a^ *p* < 0.05, ^aa^ *p* < 0.01, ^aaaa^ *p* < 0.0001 for pre-test vs. post-test, ^bbb^ *p* < 0.001, ^bbbb^ *p* < 0.0001 for post-test vs. LA-rec.

**Table 6:** WBC, LYM, MON, and GRA absolute counts corrected for Δ*PV* in the peripheral blood of the participants at the studied time points.

Variable		Younger Group		Older Group	
		Beep Test	RSA Test	BEEP TEST	RSA Test
Corrected WBC (10^9^/L)	pF ^1^	0.0015	0.0000	0.0000	0.0006
	pre-test	5.9 (5.0–7.2) ^aaa^	5.8 (5.5–6.9) ^aaaa^	5.4 (4.6–6.2) ^aaaa^	6.1 (5.5–7.2) ^aaa^
	post-test	7.8 (6.9–9.9)	10.1 (8.0–11.2) ^b^	8.7 (7.2–9.7)	8.9 (7.9–10.3)
	LA-rec	7.9 (6.5–8.7)	7.4 (6.3–9.2)	8.6 (7.1–9.5) ^cccc^	8.8 (6.7–9.7)
Corrected LYM (10^9^/L)	pF	0.0000	0.0000	0.0000	0.0000
	pre-test	2.0 (1.9–2.2) ^aa^	2.0 (1.6–2.2) ^aaa^	1.9 (1.7–2.3) ^aa^	2.2 (2.1–2.9)
	post-test	3.3 (2.6–3.9) ^bbbb^	3.8 (2.8–4.5) ^bbbb^	3.5 (2.8–4.0) ^bbbb^	3.4 (2.8–4.8) ^bbbb^
	LA-rec	1.6 (1.4–1.9)^c^	1.3 (1.2–1.8)	1.5 (1.3–1.7) ^cc^	1.5 (1.3–1.9) ^c^
Corrected MON (10^9^/L)	pF	0.0004	0.0000	0.0001	0.0000
	pre-test	0.2 (0.2–0.2) ^a^	0.2 (0.2–0.3) ^aa^	0.2 (0.1–0.2) ^aaa^	0.2 (0.2–0.2) ^aa^
	post-test	0.4 (0.3–0.4) ^bbb^	0.4 (0.3–0.6) ^bbbb^	0.4 (0.3–0.4) ^bb^	0.4 (0.4–0.5) ^bbbb^
	LA-rec	0.2 (0.1–0.2)	0.2 (0.2–0.2)	0.2 (0.2–0.2)	0.2 (0.1–0.2)
Corrected GRA (10^9^/L)	pF	0.0001	0.0002	0.0000	0.0001
	pre-test	3.6 (2.6–4.0) ^aa^	3.6 (3.0–4.4) ^aaa^	3.2 (2.8–4.0) ^aa^	3.9 (2.9–4.4) ^aa^
	post-test	4.2 (3.1–4.9)	5.6 (4.8–6.6)	4.4 (3.5–5.1)	4.9 (4.1–5.8)
	LA-rec	6.4 (4.2–7.1) ^ccc^	5.5 (3.9–7.8) ^cc^	6.1 (5.1–7.4) ^cccc^	7.0 (5.1–7.9) ^ccc^

^1^ Significance levels of differences observed between analyzed time points (pre-test vs. post-test vs. LA-rec) were assessed using Friedman’s analysis of variance for repeated measures (pF—Friedman’s ANOVA *p*-values) followed by Dunn’s post hoc test with Bonferroni correction. The table presents the median (interquartile range) of values corrected for Δ*PV*. Beep—maximal multistage 20-m shuttle run test, GRA—granulocytes, LYM—lymphocytes, MON—monocytes, RSA—reaped speed ability test, WBC—white blood cells. The analyses were performed before (baseline, pre-test) and after the effort (5 min post-effort and during LA-rec approximately 1 h after the test). Post-hoc *p* values: ^a^ *p* < 0.05, ^aa^ *p* < 0.01, ^aaa^ *p* < 0.001, ^aaaa^ *p* < 0.0001 for pre-test vs. post-test, ^b^ *p* < 0.05, ^bb^ *p* < 0.01, ^bbb^ *p* < 0.001, ^bbbb^ *p* < 0.0001, for post-test vs. LA-rec, ^c^ *p* < 0.05, ^cc^ *p* < 0.01, ^ccc^ *p* < 0.001, ^cccc^ *p* < 0.0001 for pre-test vs. LA-rec.

## Data Availability

The datasets generated and/or analyzed during the current study are available from the corresponding author on reasonable request.

## Supplementary Materials

The following supporting information can be downloaded at: https://www.mdpi.com/article/10.3390/cells12081119/s1, Figure S1: The alignment of *CCR1* amplicon to the *CCR1* reference sequence; Figure S2: The alignment of *CCR2* amplicon to the *CCR2* reference sequence; Figure S3: The alignment of *CCR3* amplicon to the *CCR3* reference sequence; Figure S4: The alignment of *CCR5* amplicon to the *CCR5* reference sequence; Figure S5: The alignment of *CXCR1* amplicon to the *CXCR1* reference sequence; Figure S6: The alignment of *CXCR2* amplicon to the *CXCR2* reference sequence; Figure S7: The alignment of *CXCR3* amplicon to the *CXCR3* reference sequence; Figure S8: The alignment of *CXCR4* amplicon to the *CXCR4* reference sequence; Figure S9: The alignment of *IL2RA* amplicon to the *IL2RA* reference sequence; Figure S10: The alignment of *IL4R* amplicon to the *IL4R* reference sequence; Figure S11: The alignment of *IL6R* amplicon to the *IL6R* reference sequence; Figure S12: The alignment of *IL10R* amplicon to the *IL10R* reference sequence; Figure S13: The alignment of *IL17RA* amplicon to the *IL17RA* reference sequence; Figure S14: The alignment of *IFNGR1* amplicon to the *IFNGR1* reference sequence; Figure S15: The alignment of *TNFR1A* amplicon to the *TNFR1A* reference sequence; Figure S16: The alignment of *TNFR1B* amplicon to the *TNFR1B* reference sequence; Figure S17: The alignment of *ACTB* amplicon to the *ACTB* reference sequence; Figure S18: The alignment of *B2M* amplicon to the *B2M* reference sequence; Figure S19: The alignment of *RACK1* amplicon to the *RACK1* reference sequence; Figure S20: Correlation between participants’ age and analyzed parameters among athletes performing Beep test. Correlation with (a) relative expression of *CXCR2* gene, (b) relative expression of *CXCR4* gene, (c) relative expression of *IL6R* gene, (d) relative expression of *IL10R* gene in pre-test time point; correlation with (e) relative expression of *IL4R* gene in post-test time point; correlation with (f) relative expression of *IFNGR1* gene, and (g) corrected LA concentration in LA-rec time point. Figure S21: Correlation between participants’ age and analyzed parameters among athletes performing RSA test. Correlation with (a) relative expression of *CCR2* gene, (b) relative expression of *IFNGR1* gene, (c) corrected lymphocyte count in pre-test time point; correlation with (d) relative expression of *CCR2* gene in post-test time point; correlation with (e) relative expression of *CCR2* gene, (f) relative expression of *IL2RA* gene, and (g) relative expression of *IL17RA* gene in LA-rec time point. Table S1: Correlation coefficients between participants’ age and corrected LA concentration, number of analyzed cells or expression of analyzed chemokine and cytokine expression at the studied time points.
