# Supplementary material for: The Impact of Different Types of Physical Effort on the Expression of Selected Chemokine and Interleukin Receptor Genes in Peripheral Blood Cells
Source: Cells. 2023 Apr 9;12(8):1119. doi: 10.3390/cells12081119 (PMC10137071; doi:10.3390/cells12081119)
Supplement: Supplementary file 1 [file cells-12-01119-s001.zip › cells-2295456-supplementary/cells-2295456_revised_supplementary-Figures.pdf]

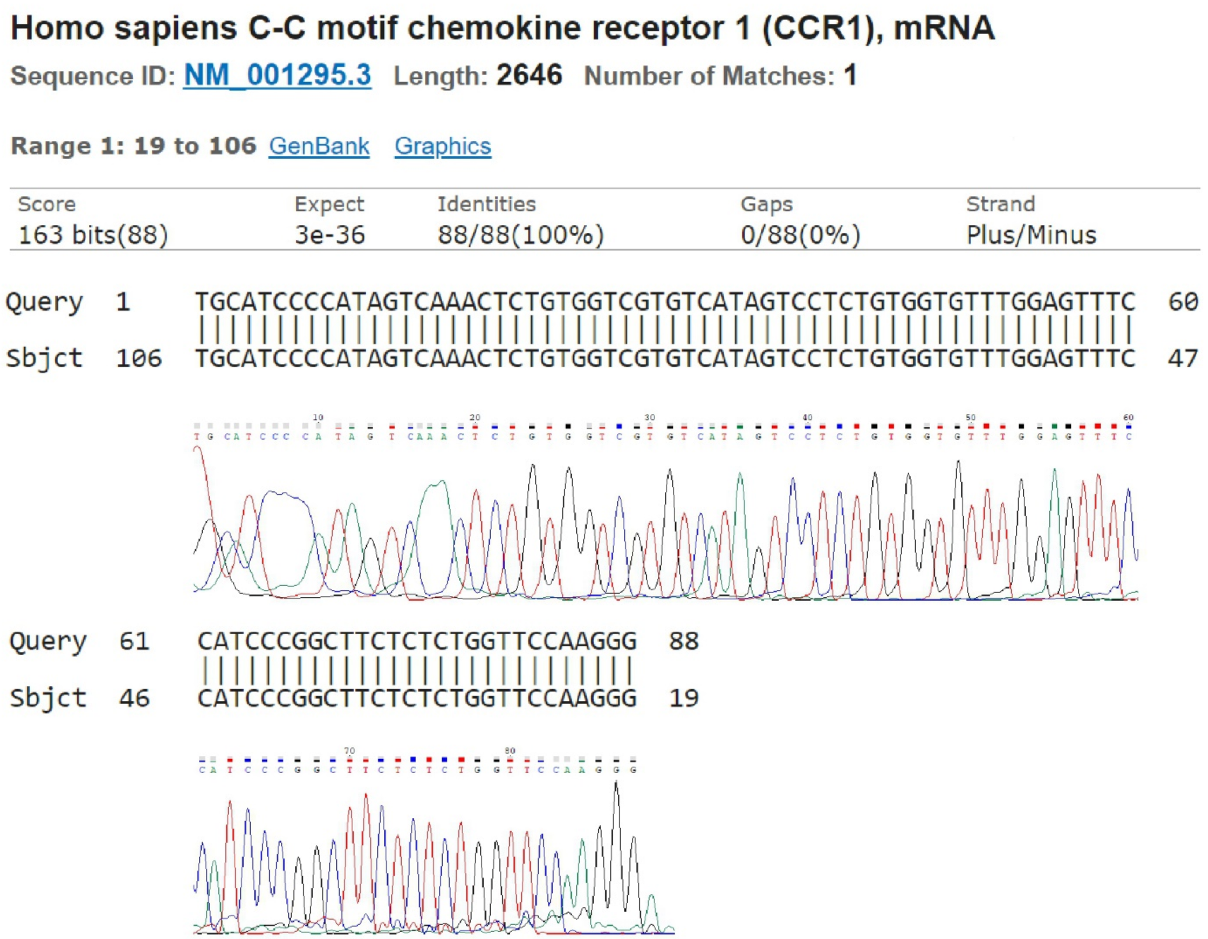

Figure S1. The alignment of *CCR1* amplicon to the *CCR1* reference sequence. The sequences were verified using Basic Local Alignment Search Tool (BLAST®; available at https://blast.ncbi.nlm.nih.gov/Blast.cgi). Chromatograms were generated using Chromas ver. 2.6.6 (Technelysium Pty Ltd., South Brisbane, Australia; available at http://technelysium.com.au/wp/chromas/).

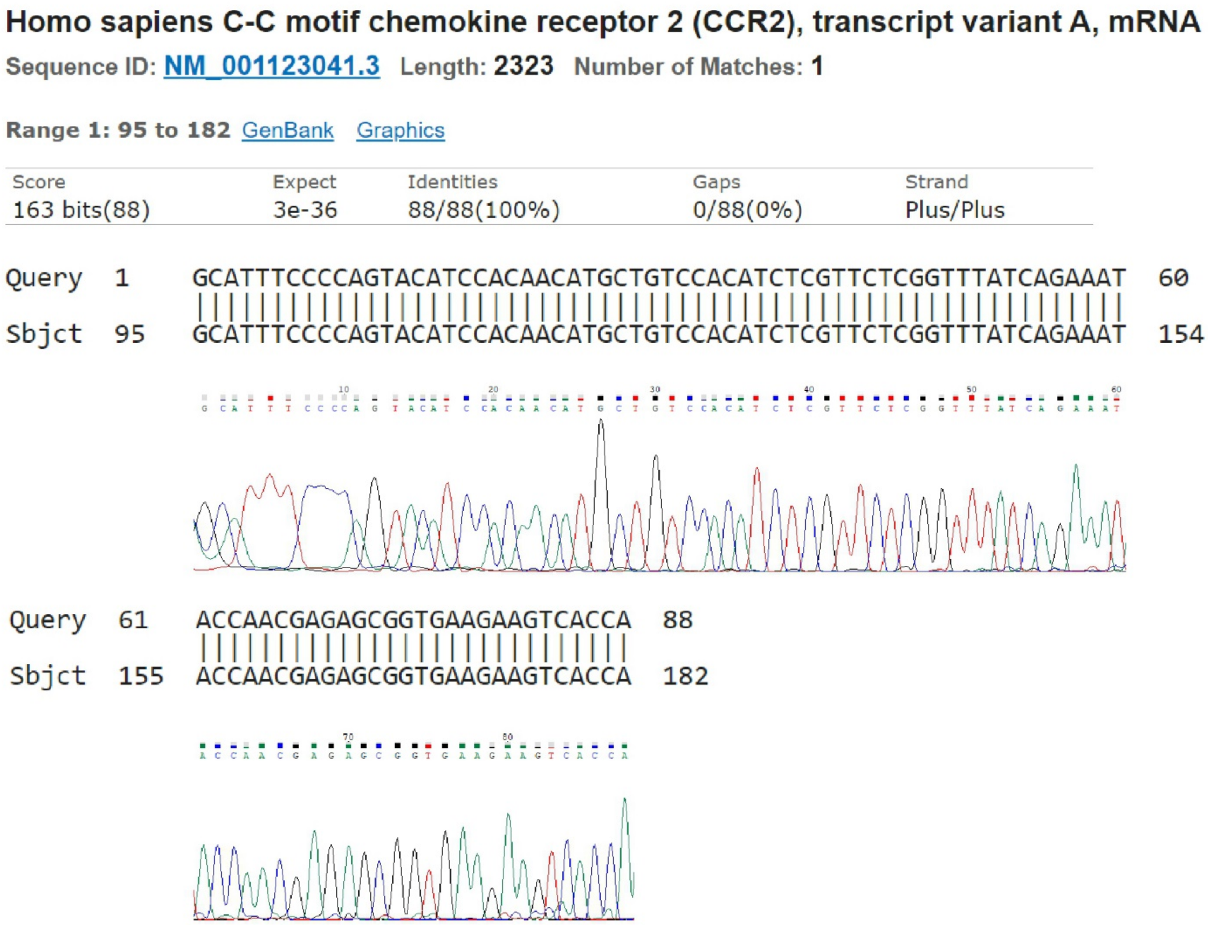

Figure S2. The alignment of *CCR2* amplicon to the *CCR2* reference sequence. For details, see legend for Figure S1.

Homo sapiens C-C motif chemokine receptor 3 (CCR3), transcript variant 1, mRNA

Sequence ID: NM_001837.4 Length: 1717 Number of Matches: 1

Range 1: 193 to 246 GenBank Graphics

Score	Expect	Identities	Gaps	Strand
93.5 bits(50)	2e-15	53/54(98%)	1/54(1%)	Plus/Plus

```
Query  8    TGAGA-CTTTGGTACCACATCCTACTATGATGACGTGGGCCTGCTCTGTGAAAA  60
            ||||| ||||||||||||||||||||||||||||||||||||||||||||||||
Sbjct  193  TGAGACCTTTGGTACCACATCCTACTATGATGACGTGGGCCTGCTCTGTGAAAA  246
```

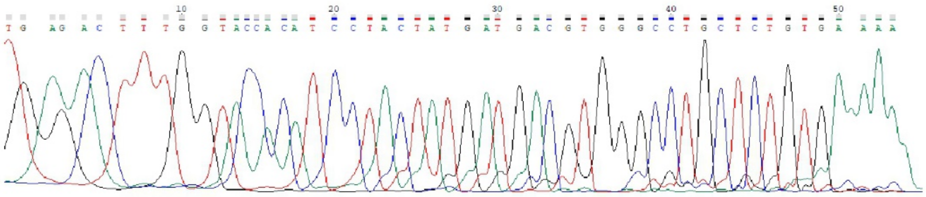

Figure S3. The alignment of *CCR3* amplicon to the *CCR3* reference sequence. For details, see legend for Figure S1.

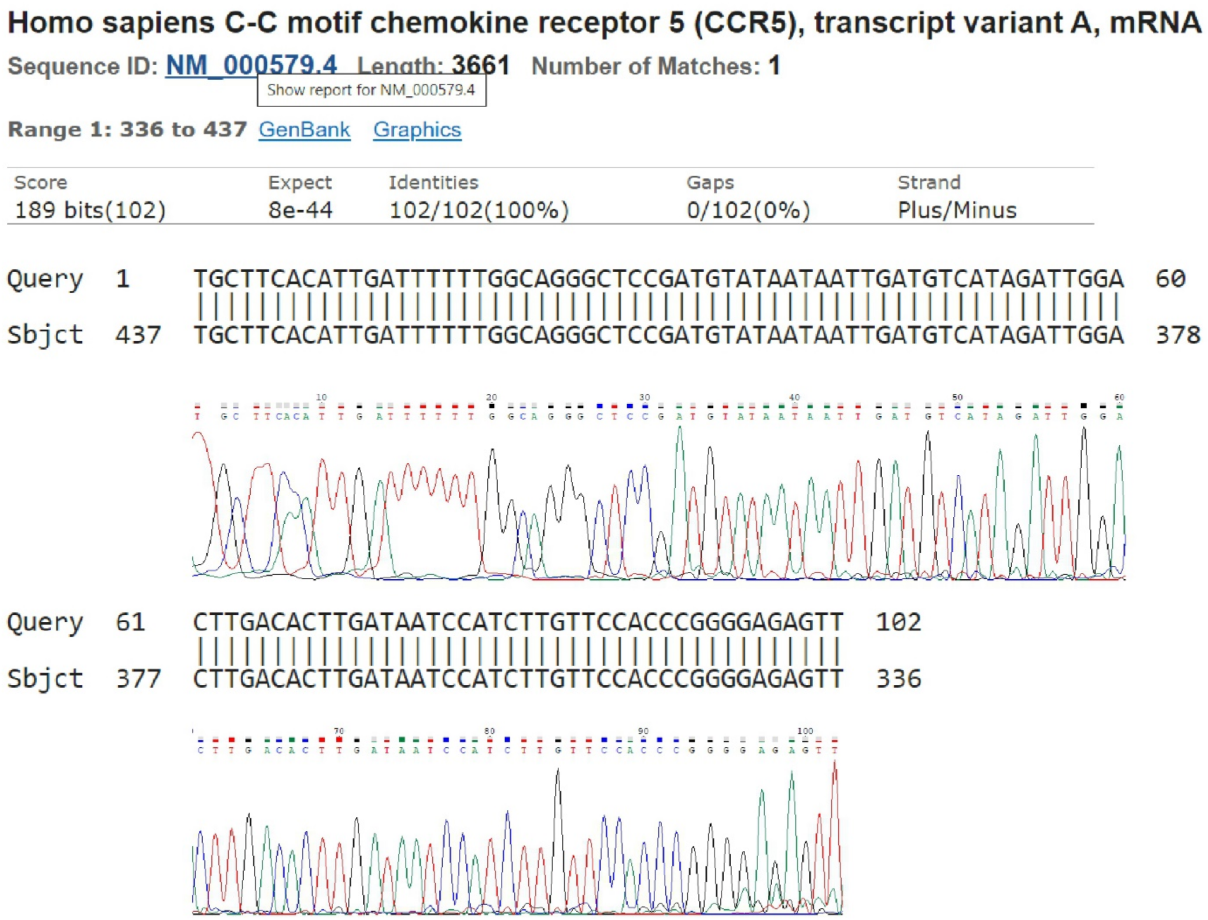

Figure S4. The alignment of *CCR5* amplicon to the *CCR5* reference sequence. For details, see legend for Figure S1.

Homo sapiens C-X-C motif chemokine receptor 1 (CXCR1), mRNA

Sequence ID: NM_000634.3 **Length:** 2459 **Number of Matches:** 1

Range 1: 67 to 176 GenBank Graphics

Score	Expect	Identities	Gaps	Strand
204 bits(110)	2e-48	110/110(100%)	0/110(0%)	Plus/Plus

```
Query  1    CATTGCTGAAACTGAAGAGGACATGTCAAATATTACAGATCCACAGATGTGGGATTTTGA  60
            ||||||||||||||||||||||||||||||||||||||||||||||||||||||||||||
Sbjct  67   CATTGCTGAAACTGAAGAGGACATGTCAAATATTACAGATCCACAGATGTGGGATTTTGA  126
```

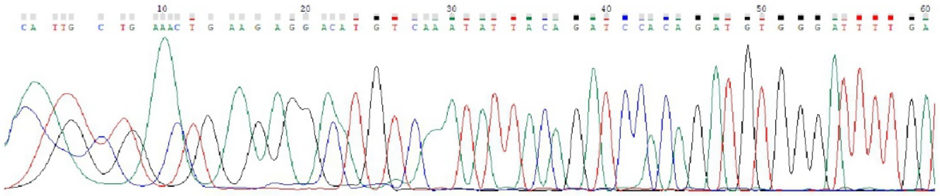

```
Query  61   TGATCTAAATTTCACTGGCATGCCACCTGCAGATGAAGATTACAGCCCCT  110
            ||||||||||||||||||||||||||||||||||||||||||||||||||
Sbjct  127  TGATCTAAATTTCACTGGCATGCCACCTGCAGATGAAGATTACAGCCCCT  176
```

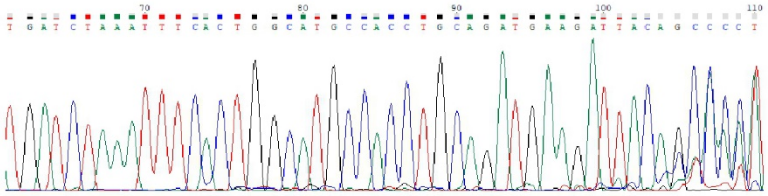

Figure S5. The alignment of *CXCR1* amplicon to the *CXCR1* reference sequence. For details, see legend for Figure S1.

Homo sapiens C-X-C motif chemokine receptor 2 (CXCR2), transcript variant 1, mRNA

Sequence ID: NM_001557.4 **Length:** 2853 **Number of Matches:** 1

Range 1: 371 to 503 GenBank Graphics

Score	Expect	Identities	Gaps	Strand
246 bits(133)	6e-61	133/133(100%)	0/133(0%)	Plus/Plus

```
Query  1    GCGACCCAGTCAGGATTTAAGTTTACCTCAAAAATGGAAGATTTTAACATGGAGAGTGAC  60
            ||||||||||||||||||||||||||||||||||||||||||||||||||||||||||||
Sbjct  371  GCGACCCAGTCAGGATTTAAGTTTACCTCAAAAATGGAAGATTTTAACATGGAGAGTGAC  430
```

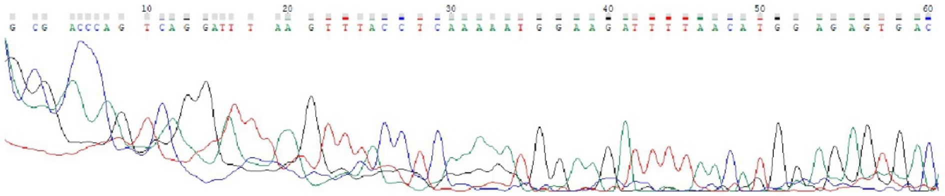

```
Query  61   AGCTTTGAAGATTTCTGGAAAGGTGAAGATCTTAGTAATTACAGTTACAGCTCTACCCTG  120
            ||||||||||||||||||||||||||||||||||||||||||||||||||||||||||||
Sbjct  431  AGCTTTGAAGATTTCTGGAAAGGTGAAGATCTTAGTAATTACAGTTACAGCTCTACCCTG  490
```

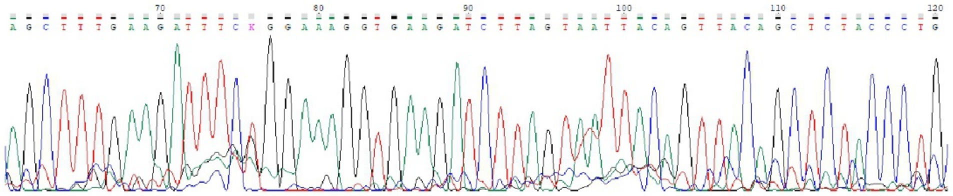

```
Query  121  CCCCCTTTTCTAC  133
            |||||||||||||
Sbjct  491  CCCCCTTTTCTAC  503
```

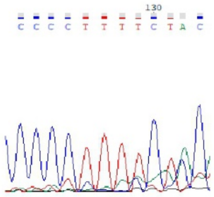

Figure S6. The alignment of *CXCR2* amplicon to the *CXCR2* reference sequence. For details, see legend for Figure S1.

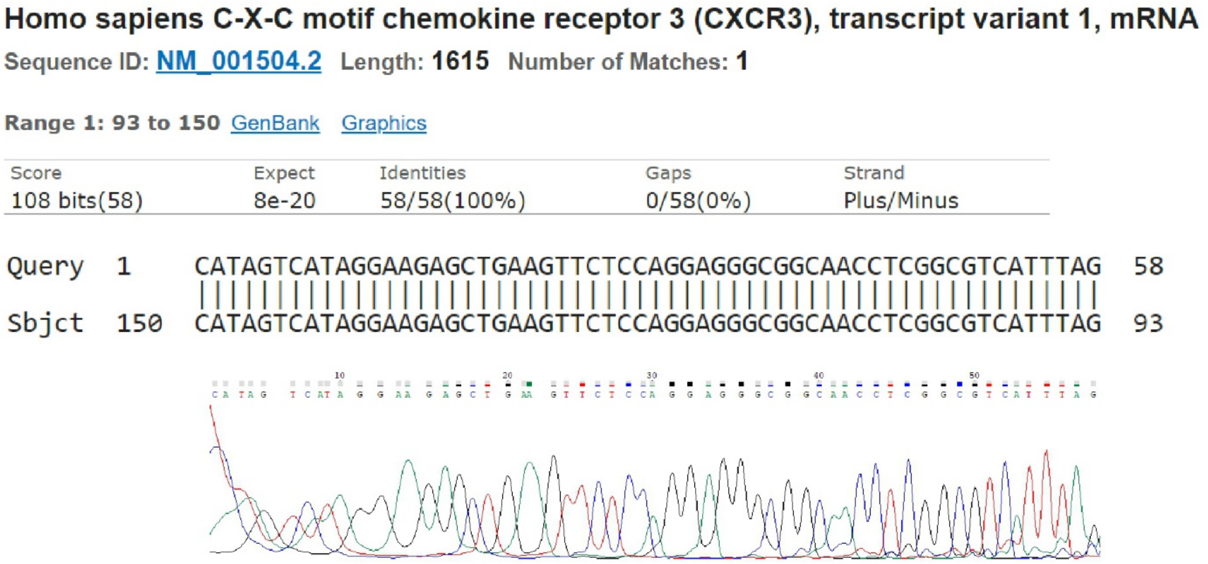

Figure S7. The alignment of *CXCR3* amplicon to the *CXCR3* reference sequence. For details, see legend for Figure S1.

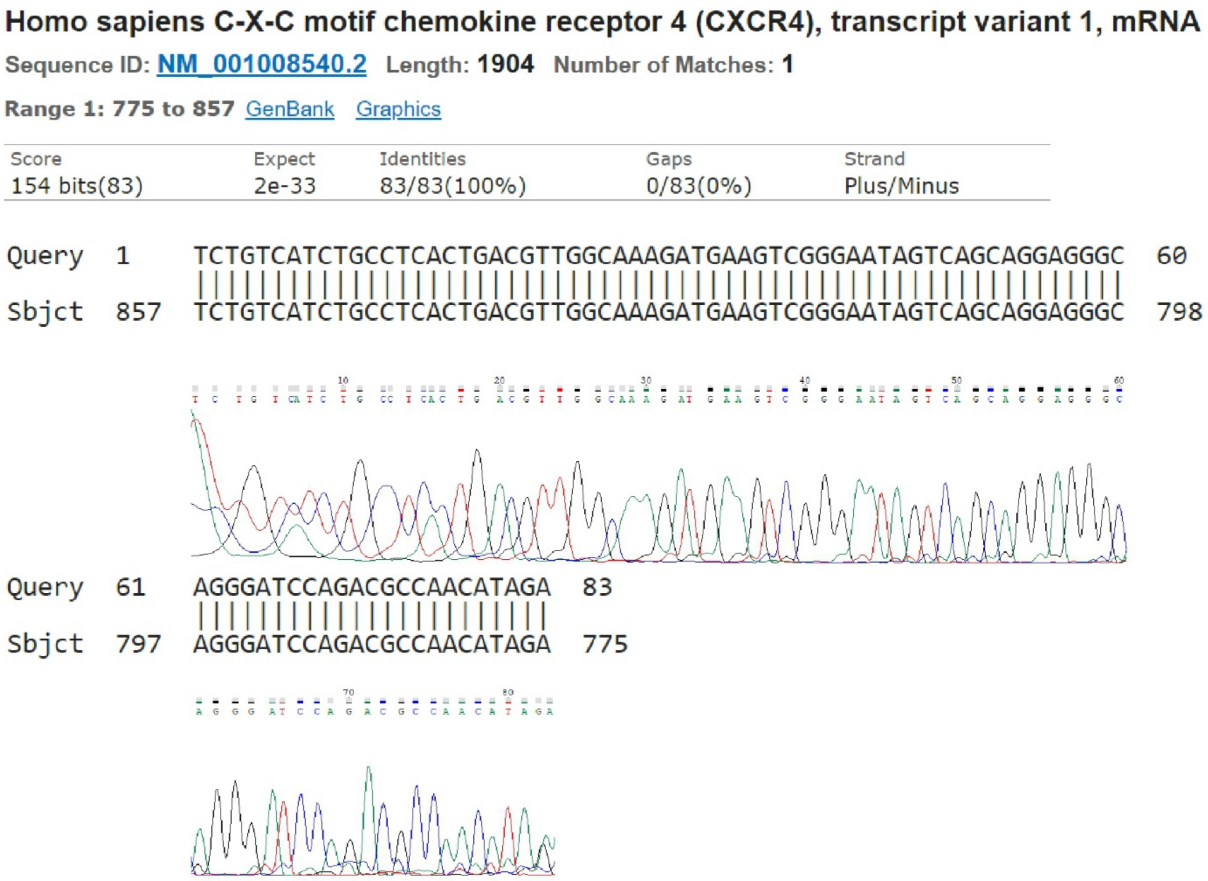

Figure S8. The alignment of *CXCR4* amplicon to the *CXCR4* reference sequence. For details, see legend for Figure S1.

Homo sapiens interleukin 2 receptor subunit alpha (IL2RA), transcript variant 1, mRNA

Sequence ID: NM_000417.3 Length: 3218 Number of Matches: 1

Range 1: 205 to 259 GenBank Graphics

Score	Expect	Identities	Gaps	Strand
102 bits(55)	3e-18	55/55(100%)	0/55(0%)	Plus/Plus

```
Query  1    AGGGTCAGGAAGATGGATTCATACCTGCTGATGTGGGGACTGCTCACGTTCATCA  55
            |||||||||||||||||||||||||||||||||||||||||||||||||||||||
Sbjct  205  AGGGTCAGGAAGATGGATTCATACCTGCTGATGTGGGGACTGCTCACGTTCATCA  259
```

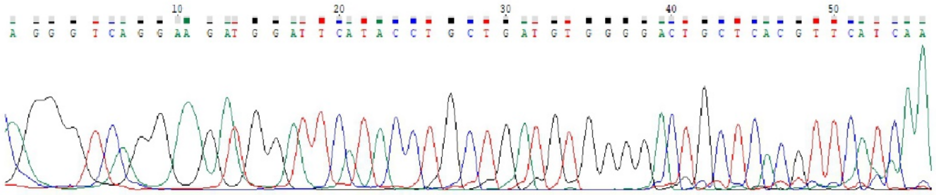

Figure S9. The alignment of *IL2RA* amplicon to the *IL2RA* reference sequence. For details, see legend for Figure S1.

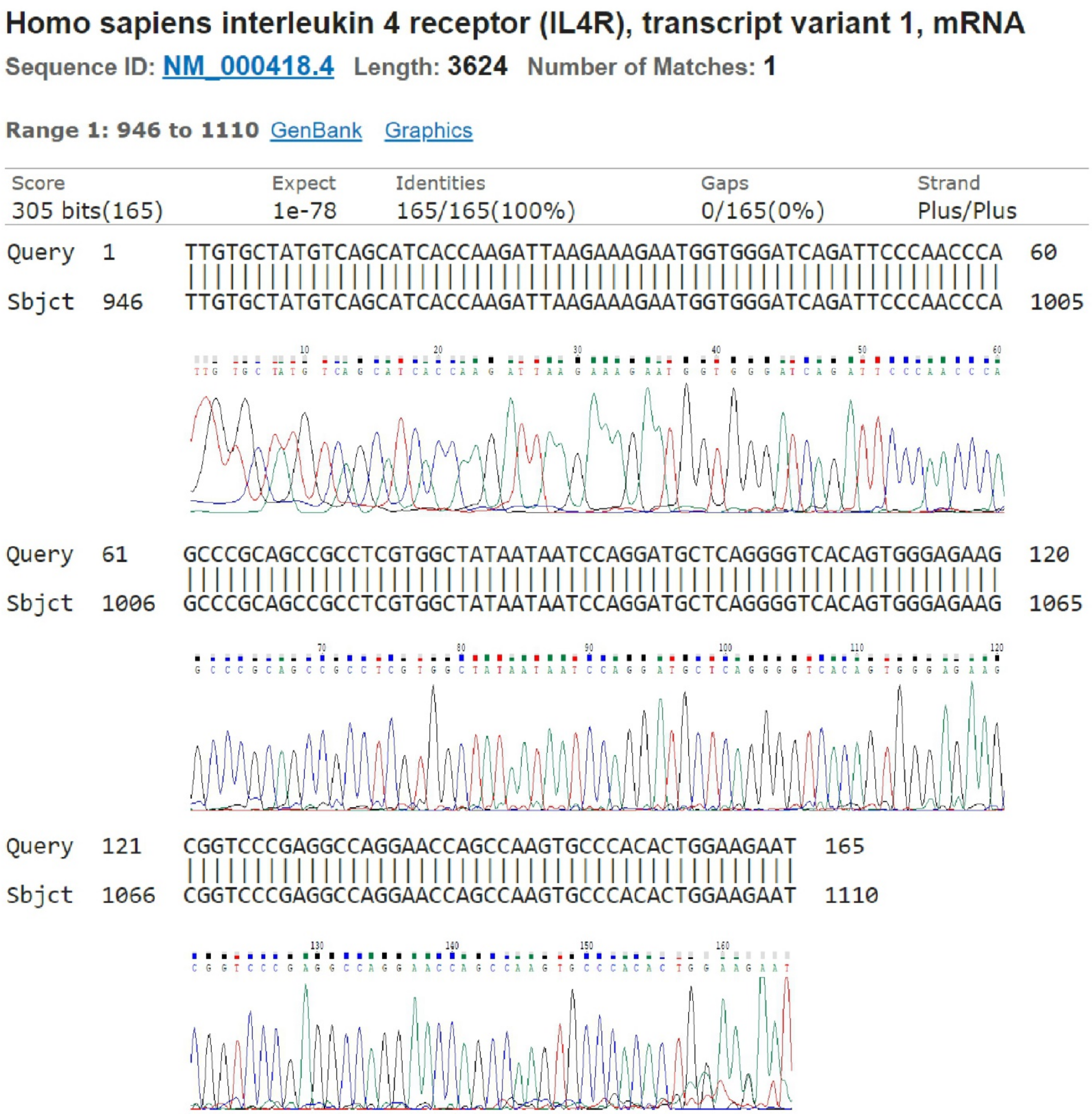

Figure S10. The alignment of *IL4R* amplicon to the *IL4R* reference sequence. For details, see legend for Figure S1.

Homo sapiens interleukin 6 receptor (IL6R), transcript variant 1, mRNA

Sequence ID: NM_000565.4 **Length:** 5764 **Number of Matches:** 1

Range 1: 988 to 1074 GenBank Graphics

Score	Expect	Identities	Gaps	Strand
161 bits(87)	1e-35	87/87(100%)	0/87(0%)	Plus/Minus

```
Query  1     TTGACCGTTCAGCCCGATATCTGAGCTCAAACCGTAGTCTGTAGAAAGATGAGTTCCAGG  60
             ||||||||||||||||||||||||||||||||||||||||||||||||||||||||||||
Sbjct  1074  TTGACCGTTCAGCCCGATATCTGAGCTCAAACCGTAGTCTGTAGAAAGATGAGTTCCAGG  1015
```

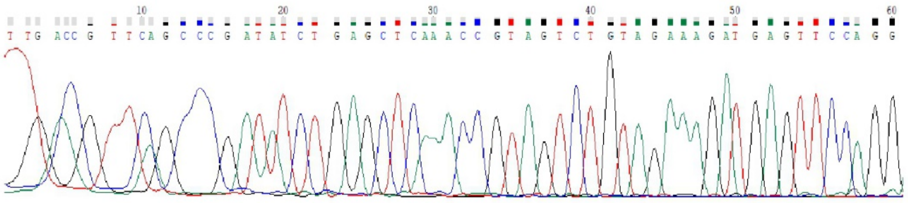

```
Query  61    AGTGGGGGTCTTGCCAGGTGACACTGA  87
             |||||||||||||||||||||||||||
Sbjct  1014  AGTGGGGGTCTTGCCAGGTGACACTGA  988
```

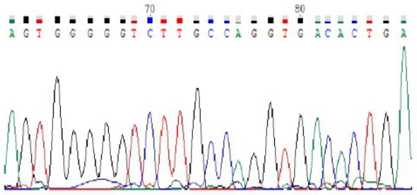

Figure S11. The alignment of *IL6R* amplicon to the *IL6R* reference sequence. For details, see legend for Figure S1.

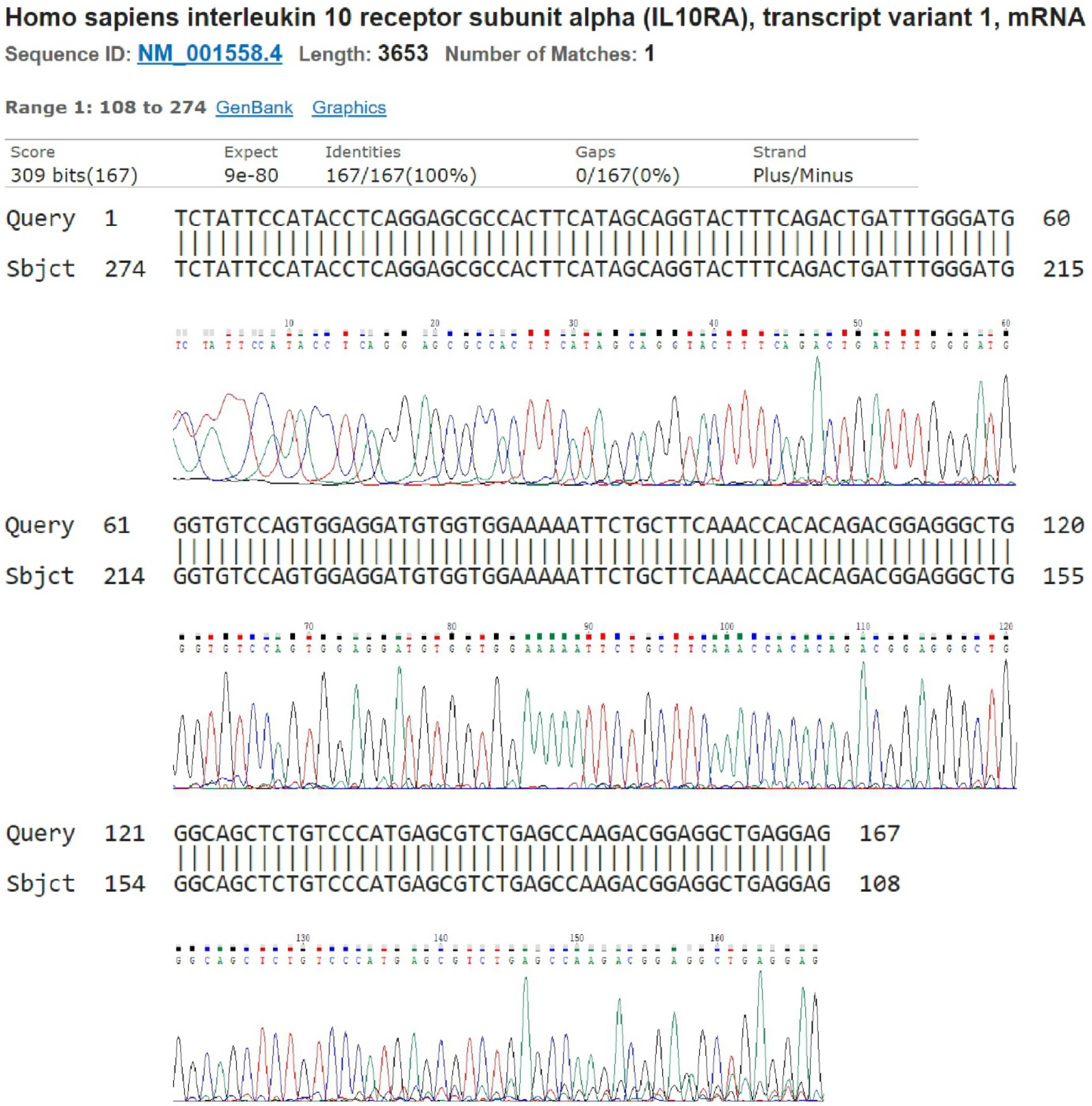

Figure S12. The alignment of *IL10R* amplicon to the *IL10R* reference sequence. For details, see legend for Figure S1.

Homo sapiens interleukin 17 receptor A (IL17RA), transcript variant 1, mRNA

Sequence ID: NM_014339.7 Length: 8566 Number of Matches: 1

Range 1: 496 to 625 GenBank Graphics

Score	Expect	Identities	Gaps	Strand
241 bits(130)	2e-59	130/130(100%)	0/130(0%)	Plus/Plus

```
Query  1    TGAGGCATCACCACAGGCGGTGGCGTTTTACCTTCAGCCACTTTGTGGTTGACCCTGACC  60
            ||||||||||||||||||||||||||||||||||||||||||||||||||||||||||||
Sbjct  496  TGAGGCATCACCACAGGCGGTGGCGTTTTACCTTCAGCCACTTTGTGGTTGACCCTGACC  555
```

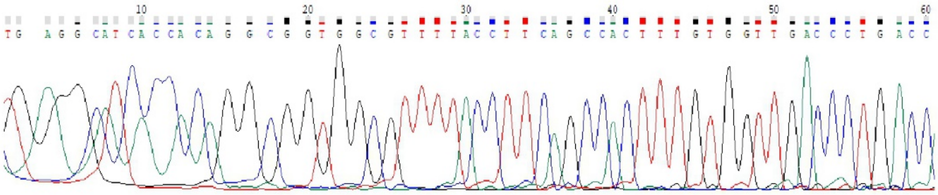

```
Query  61   AGGAATATGAGGTGACCGTTCACCACCTGCCCAAGCCCATCCCTGATGGGGACCCAAACC  120
            ||||||||||||||||||||||||||||||||||||||||||||||||||||||||||||
Sbjct  556  AGGAATATGAGGTGACCGTTCACCACCTGCCCAAGCCCATCCCTGATGGGGACCCAAACC  615
```

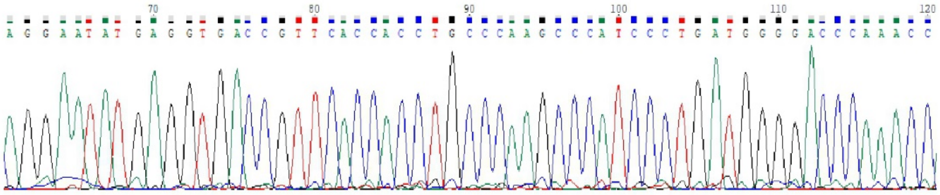

```
Query  121  ACCAGTCCAA  130
            ||||||||||
Sbjct  616  ACCAGTCCAA  625
```

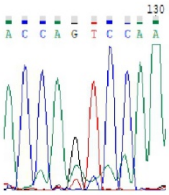

Figure S13. The alignment of *IL17RA* amplicon to the *IL17RA* reference sequence. For details, see legend for Figure S1.

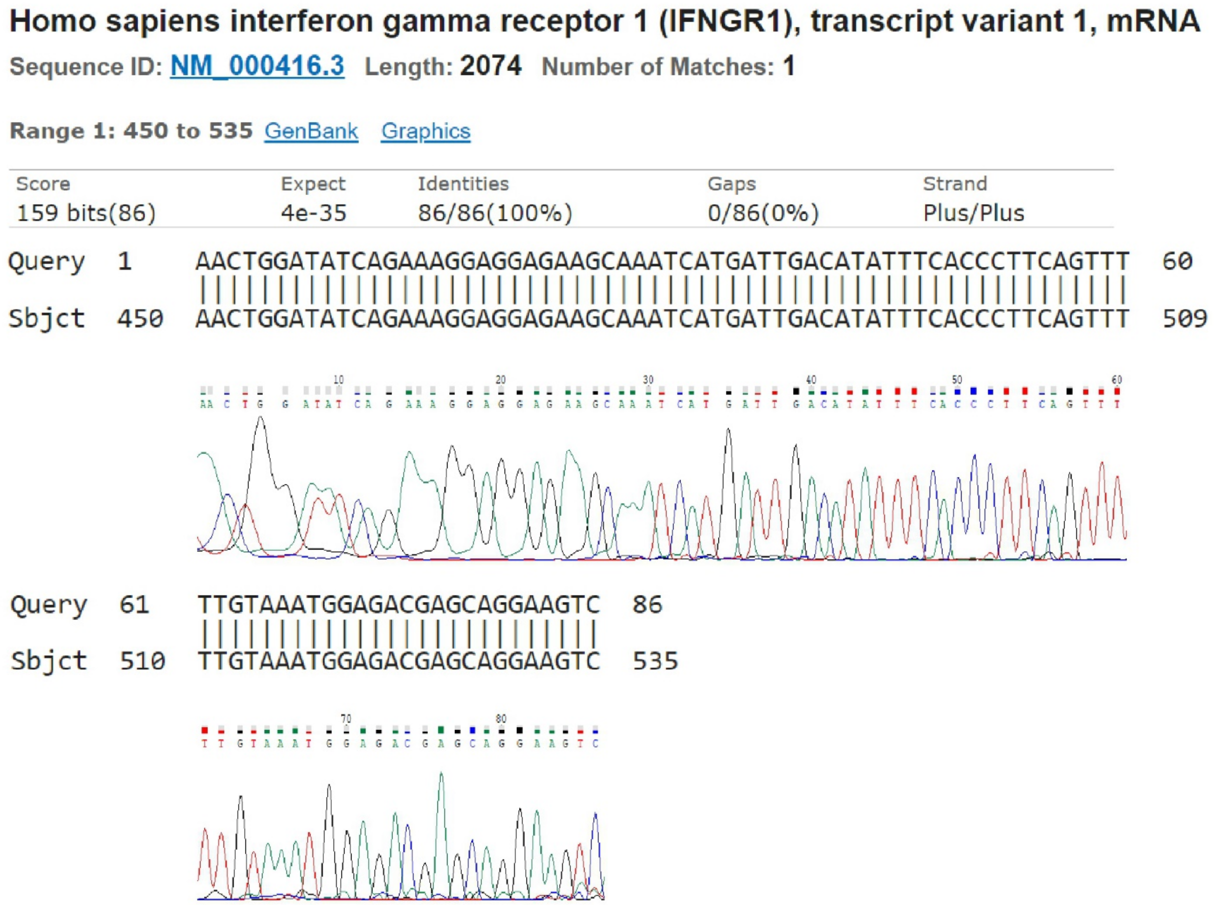

Figure S14. The alignment of *IFNGR1* amplicon to the *IFNGR1* reference sequence. For details, see legend for Figure S1.

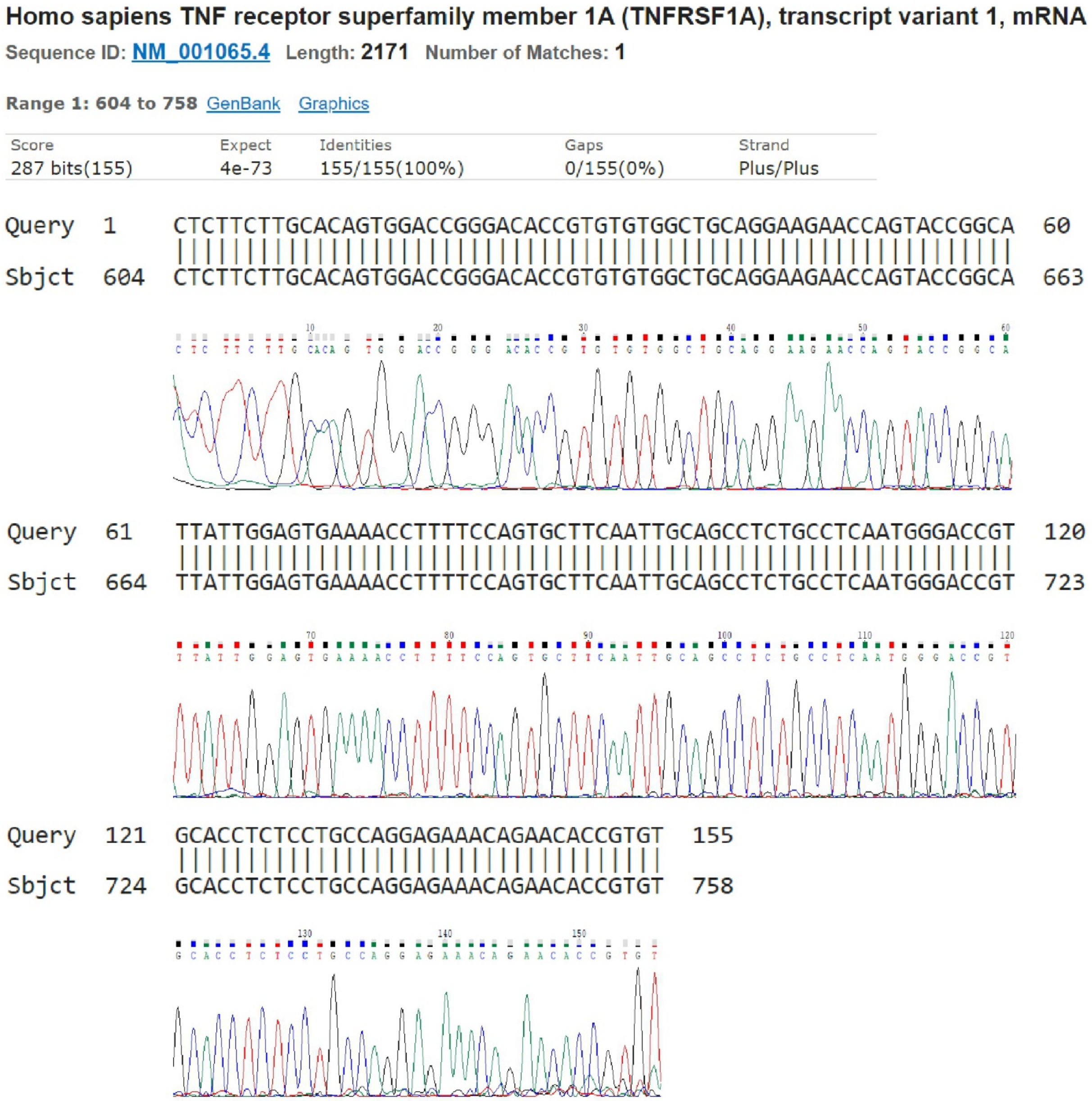

Figure S15. The alignment of *TNFR1A* amplicon to the *TNFR1A* reference sequence. For details, see legend for Figure S1.

Homo sapiens TNF receptor superfamily member 1B (TNFRSF1B), mRNA

Sequence ID: NM_001066.3 Length: 3687 Number of Matches: 1

Range 1: 183 to 389 GenBank Graphics

Score	Expect	Identities	Gaps	Strand
377 bits(204)	3e-100	206/207(99%)	0/207(0%)	Plus/Minus

```
Query  1    ACAGCTCAAGCACTCGGGAACCCAGTTCCAGAGCTGGGTGTATGTGCTGTCCTCACAGGA  60
            ||||||||||||||||||||||||||||||||||||||||||||||||||||||||||||
Sbjct  389  ACAGCTCAAGCACTCGGGAACCCAGTTCCAGAGCTGGGTGTATGTGCTGTCCTCACAGGA  330
```

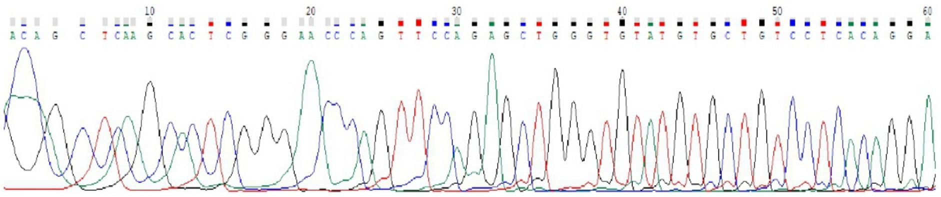

```
Query  61   GTCACACACGGTGTCCGAGGTCTTGGTACAGAAGACTTTTGCATGTTGGCCCGGCGAGCA  120
            ||||||||||||||||||||||||||||||||||||||||||||||||||||||||||||
Sbjct  329  GTCACACACGGTGTCCGAGGTCTTGGTACAGAAGACTTTTGCATGTTGGCCCGGCGAGCA  270
```

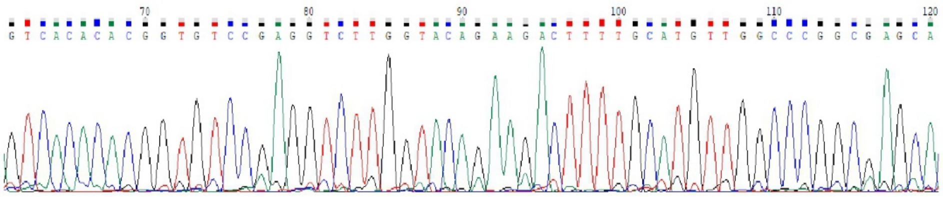

```
Query  121  CTTGCTGCAGCACATCTGAGCTGTCTGGTCATAGTATTCTCTGAGCCGGCATGTGCTCCC  180
             |||||||||||||||||||||||||||||||||||||||||||||||||||||||||||
Sbjct  269  TTTGCTGCAGCACATCTGAGCTGTCTGGTCATAGTATTCTCTGAGCCGGCATGTGCTCCC  210
```

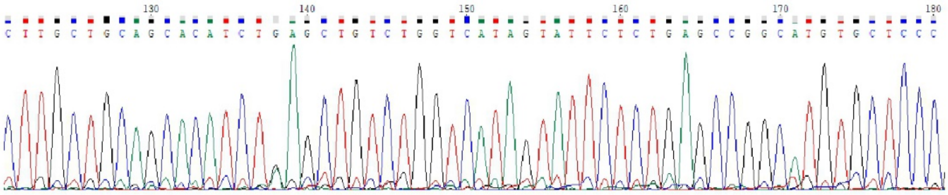

```
Query  181  GGGCTCCGGGGCGTAGGGTGTAAATGC  207
            |||||||||||||||||||||||||||
Sbjct  209  GGGCTCCGGGGCGTAGGGTGTAAATGC  183
```

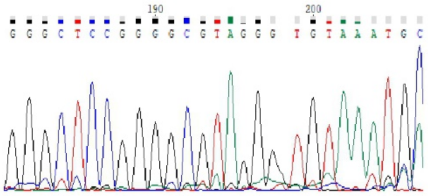

Figure S16. The alignment of *TNFR1B* amplicon to the *TNFR1B* reference sequence. For details, see legend for Figure S1.

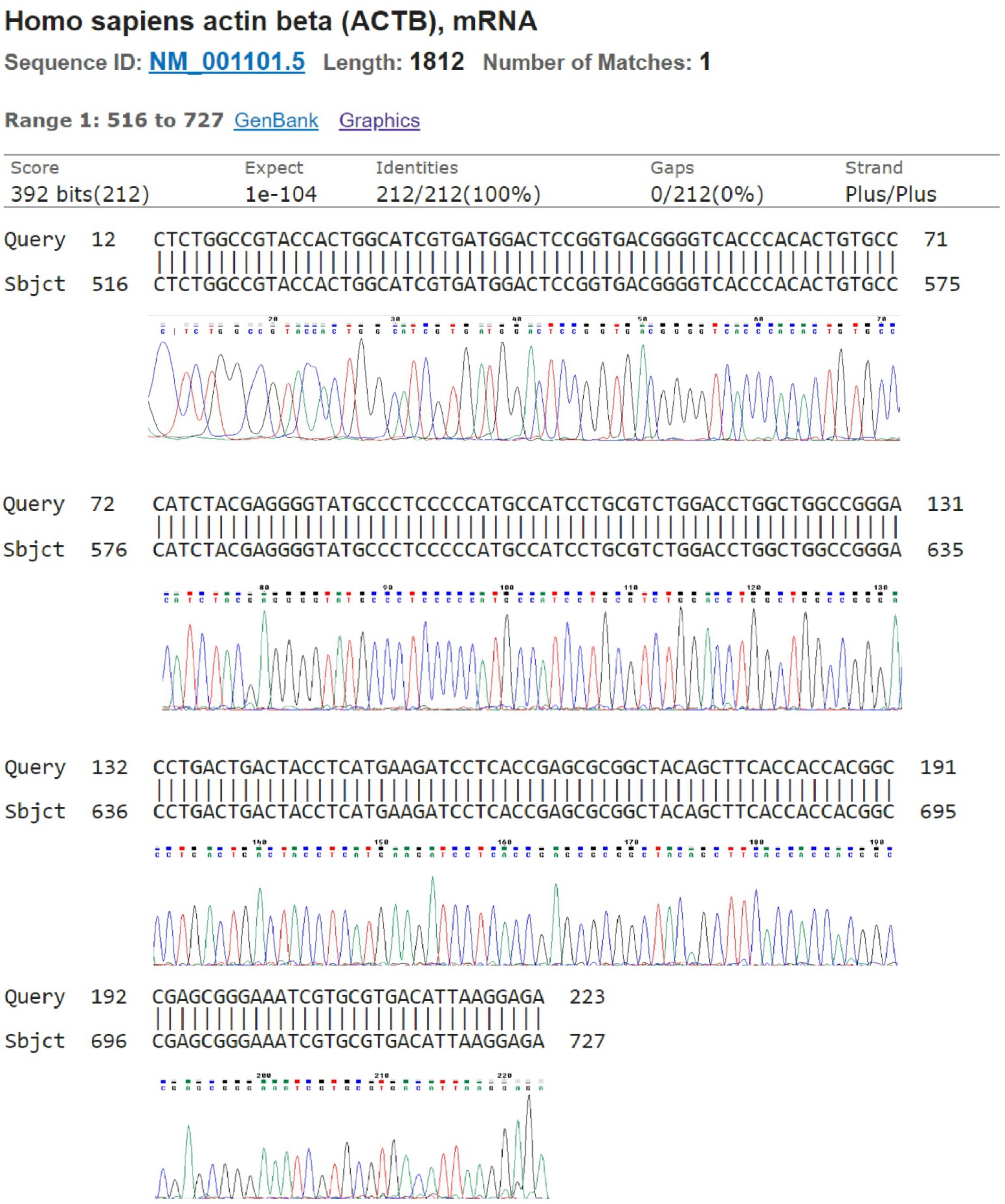

Figure S17. The alignment of *ACTB* amplicon to the *ACTB* reference sequence. For details, see legend for Figure S1.

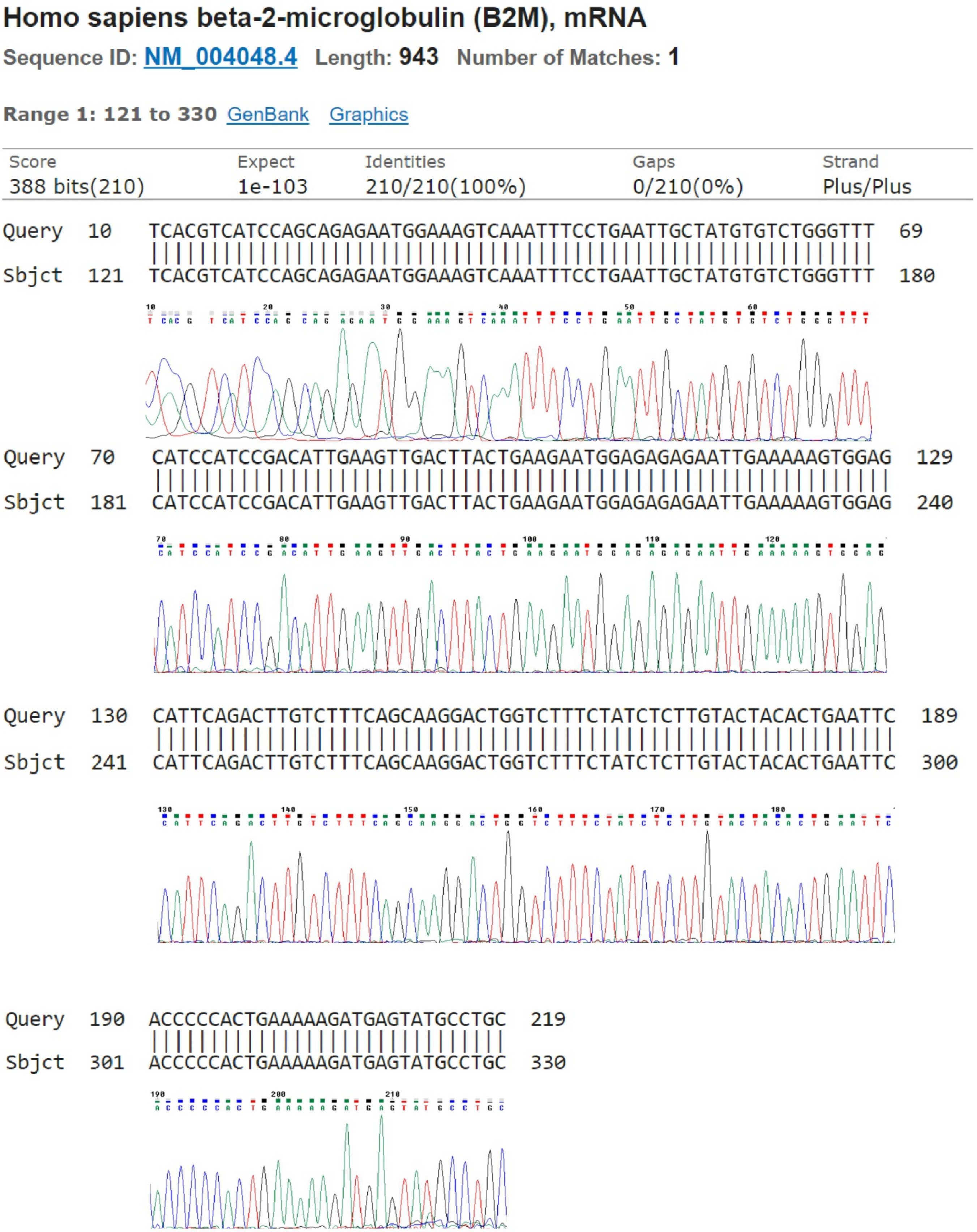

Figure S18. The alignment of *B2M* amplicon to the *B2M* reference sequence. For details, see legend for Figure S1.

Homo sapiens receptor for activated C kinase 1 (RACK1), mRNA

Sequence ID: NM_006098.5 Length: 1140 Number of Matches: 1

Range 1: 434 to 640 GenBank Graphics

Score	Expect	Identities	Gaps	Strand
383 bits(207)	6e-102	207/207(100%)	0/207(0%)	Plus/Plus

```
Query  1    AGTGTGGCCTTCTCCTCTGACAACCGGCAGATTGTCTCTGGATCTCGAGATAAAACCATC  60
            ||||||||||||||||||||||||||||||||||||||||||||||||||||||||||||
Sbjct  434  AGTGTGGCCTTCTCCTCTGACAACCGGCAGATTGTCTCTGGATCTCGAGATAAAACCATC  493
```

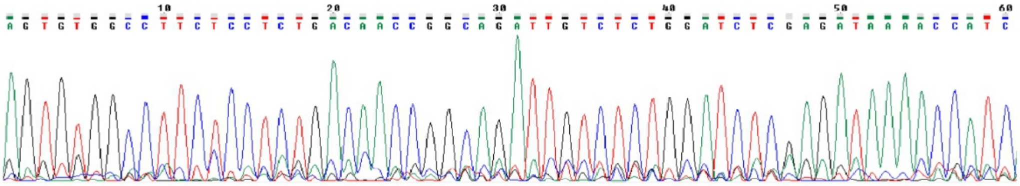

```
Query  61   AAGCTATGGAATACCCTGGGTGTGTGCAAATACACTGTCCAGGATGAGAGCCACTCAGAG  120
            ||||||||||||||||||||||||||||||||||||||||||||||||||||||||||||
Sbjct  494  AAGCTATGGAATACCCTGGGTGTGTGCAAATACACTGTCCAGGATGAGAGCCACTCAGAG  553
```

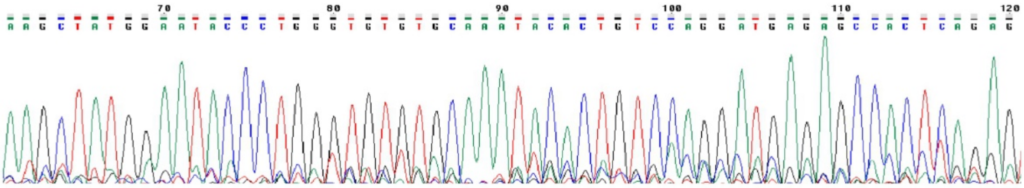

```
Query  121  TGGGTGTCTTGTGTCCGCTTCTCGCCCAACAGCAGCAACCCTATCATCGTCTCCTGTGGC  180
            ||||||||||||||||||||||||||||||||||||||||||||||||||||||||||||
Sbjct  554  TGGGTGTCTTGTGTCCGCTTCTCGCCCAACAGCAGCAACCCTATCATCGTCTCCTGTGGC  613
```

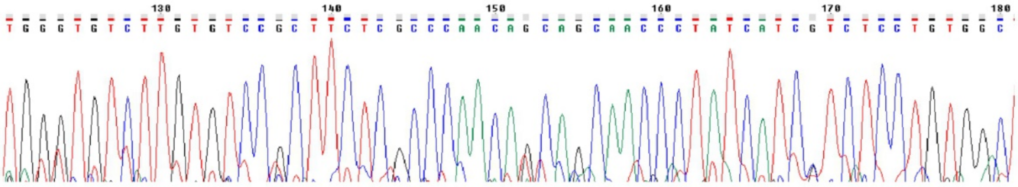

```
Query  181  TGGGACAAGCTGGTCAAGGTATGGAAC  207
            |||||||||||||||||||||||||||
Sbjct  614  TGGGACAAGCTGGTCAAGGTATGGAAC  640
```

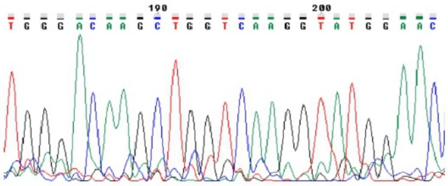

Figure S19. The alignment of *RACK1* amplicon to the *RACK1* reference sequence. For details, see legend for Figure S1.

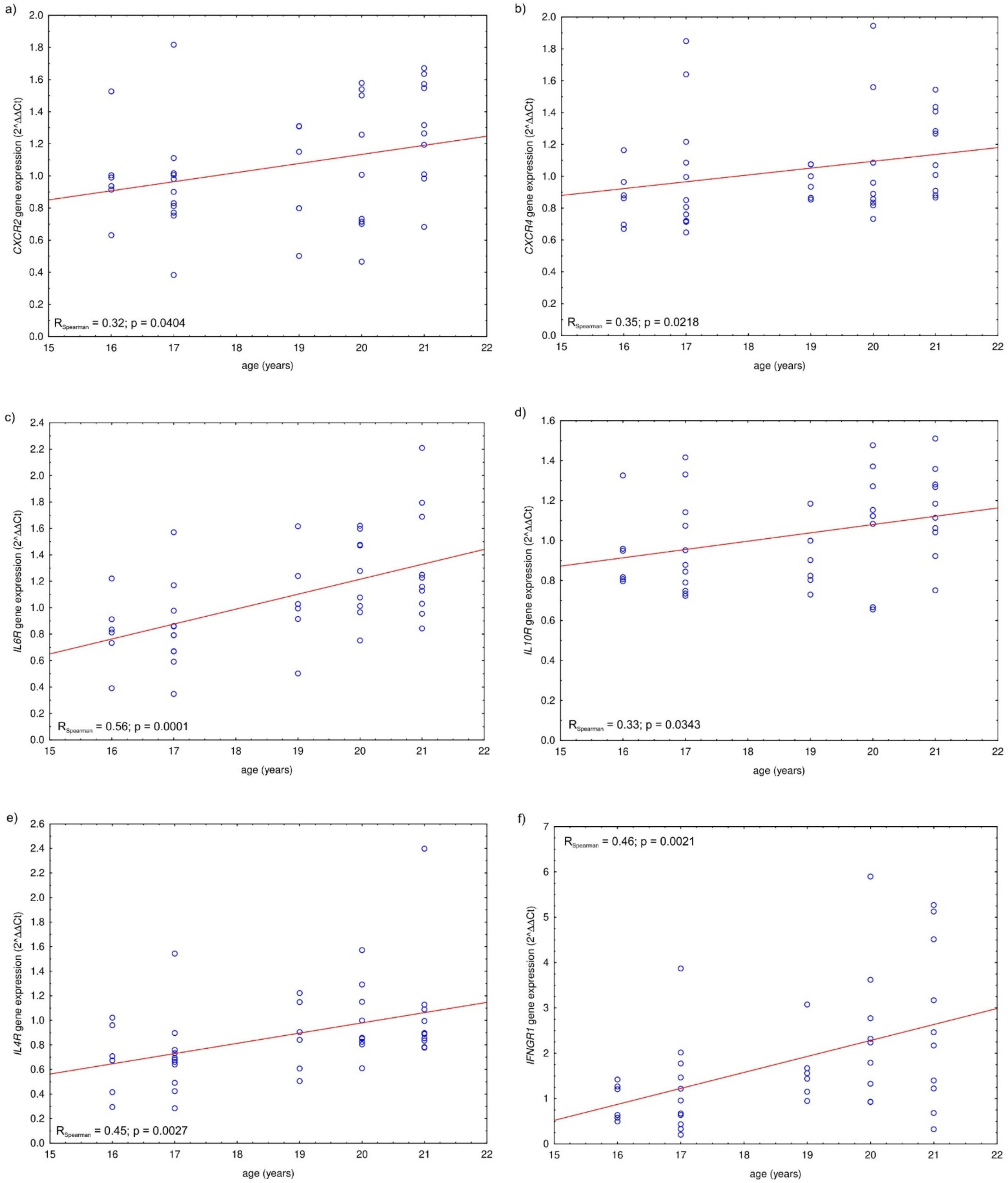
a)
CXCR2 gene expression (2^ΔΔCt)
R Spearman = 0.32; p = 0.0404
age (years)
b)
CXCR4 gene expression (2^ΔΔCt)
R Spearman = 0.35; p = 0.0218
age (years)
c)
IL6R gene expression (2^ΔΔCt)
R Spearman = 0.56; p = 0.0001
age (years)
d)
IL10R gene expression (2^ΔΔCt)
R Spearman = 0.33; p = 0.0343
age (years)
e)
IL4R gene expression (2^ΔΔCt)
R Spearman = 0.45; p = 0.0027
age (years)
f)
IFNGR1 gene expression (2^ΔΔCt)
R Spearman = 0.46; p = 0.0021
age (years)

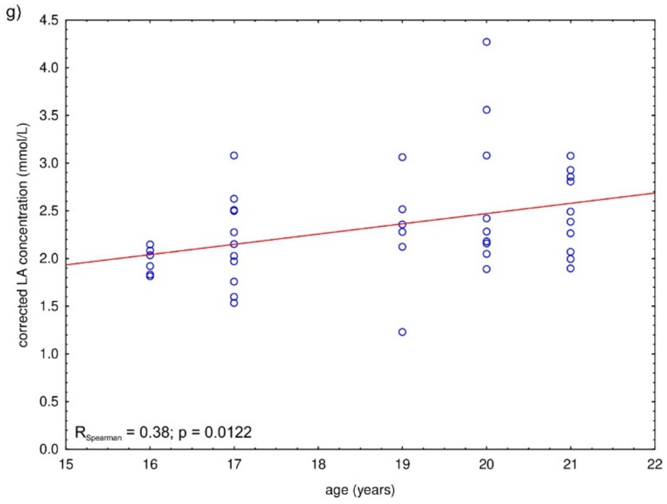

Figure S20. Correlation between participants' age and analyzed parameters among athletes performing Beep test. Correlation with (a) relative expression of *CXCR2* gene, (b) relative expression of *CXCR4* gene, (c) relative expression of *IL6R* gene, (d) relative expression of *IL10R* gene in pre-test time point; correlation with (e) relative expression of *IL4R* gene in post-test time point; correlation with (f) relative expression of *IFNGR1* gene, and g) corrected LA concentration in LA-rec time point.

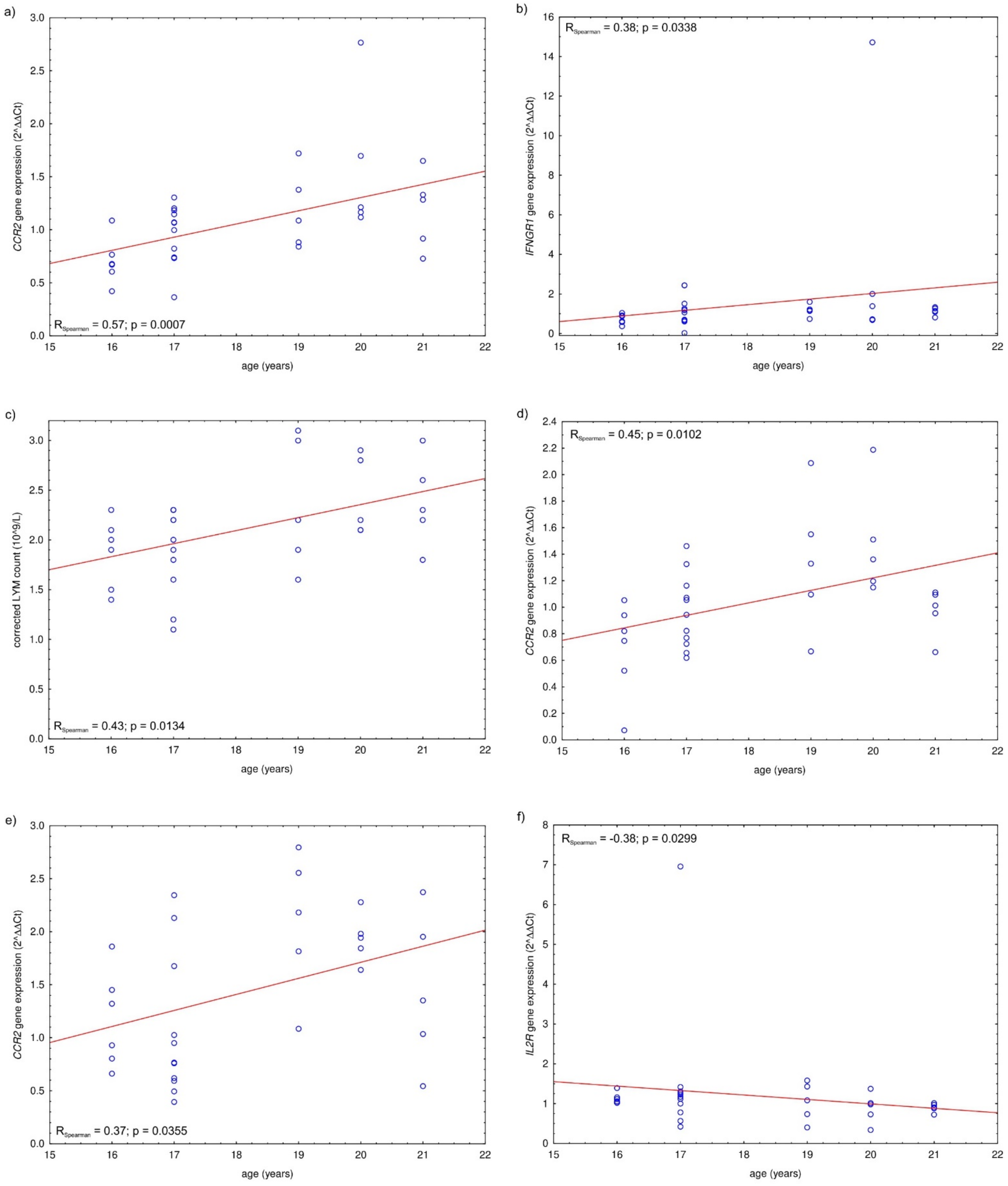

a)
CCR2 gene expression (2^ΔΔCt)
RSpearman = 0.57; p = 0.0007
age (years)
b)
IFNGR1 gene expression (2^ΔΔCt)
RSpearman = 0.38; p = 0.0338
age (years)
c)
corrected LYM count (10^9/L)
RSpearman = 0.43; p = 0.0134
age (years)
d)
CCR2 gene expression (2^ΔΔCt)
RSpearman = 0.45; p = 0.0102
age (years)
e)
CCR2 gene expression (2^ΔΔCt)
RSpearman = 0.37; p = 0.0355
age (years)
f)
IL2R gene expression (2^ΔΔCt)
RSpearman = -0.38; p = 0.0299
age (years)

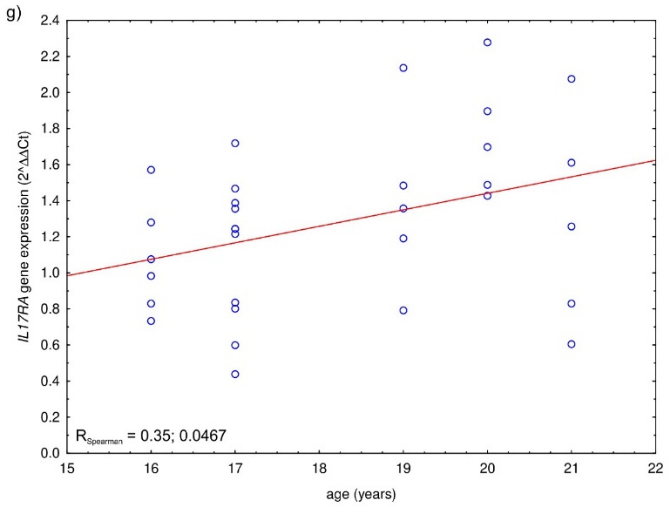

Figure S21. Correlation between participants' age and analyzed parameters among athletes performing RSA test. Correlation with (a) relative expression of *CCR2* gene, (b) relative expression of *IFNGR1* gene, (c) corrected lymphocyte count in pre-test time point; correlation with (d) relative expression of *CCR2* gene in post-test time point; correlation with correlation with (e) relative expression of *CCR2* gene, (f) relative expression of *IL2RA* gene, and (g) relative expression of *IL17RA* gene in LA-rec time point.
