# Supplementary material for: The Impact of Different Types of Physical Effort on the Expression of Selected Chemokine and Interleukin Receptor Genes in Peripheral Blood Cells
Source: Cells. 2023 Apr 9;12(8):1119. doi: 10.3390/cells12081119 (PMC10137071; doi:10.3390/cells12081119)
Supplement: Supplementary file 1 [file cells-12-01119-s001.zip › cells-2295456-supplementary/cells-2295456_revised_supplementary-TableS1.pdf]

Supplementary material

Table S1. Correlations coefficients between participants' age and corrected LA concentration, number of analyzed cells or expression of analyzed chemokine and cytokine expression at the studied time points.

**The correlation between participants' age (years) and** [1]		**Beep test**		**RSA test**	
		R spearman	p	R spearman	p
Corrected LA (mmol/L)	pre-test	-0.04	0.8095	-0.21	0.2432
	post-test	0.02	0.9210	-0.03	0.8801
	LA-rec	0.38	0.0122	-0.11	0.5549
Corrected WBC (10^9/L)	pre-test	-0.14	0.3701	0.15	0.3992
	post-test	0.17	0.2781	-0.07	0.7124
	LA-rec	0.10	0.5418	0.18	0.3223
Corrected LYM (10^9/L)	pre-test	-0.16	0.3118	0.43	0.0134
	post-test	0.15	0.3312	0.12	0.5027
	LA-rec	-0.16	0.3249	0.14	0.4425
Corrected MON (10^9/L)	pre-test	-0.21	0.1859	-0.01	0.9426
	post-test	0.10	0.5266	-0.01	0.9346
	LA-rec	-0.01	0.9609	0.01	0.9517
Corrected GRA (10^9/L)	pre-test	-0.04	0.7853	0.08	0.6483
	post-test	0.24	0.1181	-0.20	0.2809
	LA-rec	0.10	0.5362	0.10	0.5951
CCR1 (2^ΔΔCt)	pre-test	0.08	0.6286	0.15	0.4063
	post-test	0.22	0.1534	0.12	0.5060
	LA-rec	-0.09	0.5649	0.37	0.0355
CCR2 (2^ΔΔCt)	pre-test	0.14	0.3672	0.57	0.0007
	post-test	0.21	0.1811	0.45	0.0102
	LA-rec	0.09	0.5732	0.17	0.3359
CCR3 (2^ΔΔCt)	pre-test	-0.07	0.6480	0.29	0.1031
	post-test	-0.14	0.3901	0.29	0.1093
	LA-rec	-0.22	0.1610	-0.12	0.5174
CCR5 (2^ΔΔCt)	pre-test	-0.10	0.5092	0.06	0.7410
	post-test	0.07	06541	-0.13	0.4876
	LA-rec	-0.25	0.1057	0.30	0.0901
CXCR1 (2^ΔΔCt)	pre-test	0.17	0.2906	0.25	0.1716
	post-test	-0.05	0.7709	0.10	0.5936
	LA-rec	-0.02	0.8975	0.07	0.6986
CXCR2 (2^ΔΔCt)	pre-test	0.32	0.0404	0.27	0.1331
	post-test	0.15	0.3354	0.17	0.3465
	LA-rec	0.30	0.0544	0.07	0.6971
CXCR3 (2^ΔΔCt)	pre-test	0.21	0.1910	0.20	0.2828
	post-test	0.25	0.1162	-0.05	0.7880
	LA-rec	0.03	0.8233	0.34	0.0539
CXCR4 (2^ΔΔCt)	pre-test	0.35	0.0218	0.09	0.6409
	post-test	0.18	0.2630	-0.04	0.8086
	LA-rec	0.04	0.2642	-0.38	0.0299
IL2RA (2^ΔΔCt)	pre-test	0.10	0.5089	0.26	0.1519
	post-test	-0.09	0.5643	0.04	0.8359
	LA-rec	-0.09	0.5526	-0.20	0.2629
IL4R (2^ΔΔCt)	pre-test	0.28	0.0702	0.07	0.6835

	post-test	0.45	0.0027	0.11	0.5397
	LA-rec	0.14	0.3783	0.27	0.1297
	pre-test	0.56	0.0001	0.29	0.1122
IL6R (2^ΔΔCt)	post-test	0.29	0.0579	-0.003	0.9869
	LA-rec	0.25	0.1147	-0.28	0.1188
	pre-test	0.33	0.0343	0.22	0.2155
IL10R (2^ΔΔCt)	post-test	0.22	0.1622	-0.14	0.4358
	LA-rec	-0.17	0.2692	0.35	0.0467
	pre-test	0.01	0.9405	-0.09	0.6058
IL17RA (2^ΔΔCt)	post-test	0.02	0.8876	0.15	0.4039
	LA-rec	0.14	0.3780	0.14	0.4296
	pre-test	0.17	0.2935	0.38	0.0338
IFNGR1 (2^ΔΔCt)	post-test	0.28	0.0684	0.21	0.2453
	LA-rec	0.46	0.0021	-0.16	0.3651
	pre-test	0.20	0.1962	0.12	0.5160
TNFR1A (2^ΔΔCt)	post-test	0.12	0.4643	-0.06	0.7294
	LA-rec	0.11	0.4971	0.07	0.6729
	pre-test	0.11	0.4945	0.28	0.1140
TNFR1B (2^ΔΔCt)	post-test	0.24	0.1346	0.21	0.2393
	LA-rec	0.03	0.8662	-0.14	0.4139

[1] The correlations between participants' age and corrected LA concentration, number of analyzed cells or expression of analyzed chemokine and cytokine expression, respectively were assessed using Spear-man's rank correlation coefficient determination.
